# Structural insights into the electron/proton transfer pathways in the quinol:fumarate reductase from *Desulfovibrio gigas*

**DOI:** 10.1038/s41598-018-33193-5

**Published:** 2018-10-08

**Authors:** Hong-Hsiang Guan, Yin-Cheng Hsieh, Pei-Ju Lin, Yen-Chieh Huang, Masato Yoshimura, Li-Ying Chen, Shao-Kang Chen, Phimonphan Chuankhayan, Chien-Chih Lin, Nai-Chi Chen, Atsushi Nakagawa, Sunney I. Chan, Chun-Jung Chen

**Affiliations:** 1 Life Science Group, Scientific Research Division, National Synchrotron Radiation Research Center, Hsinchu, 30076 Taiwan; 2 Institute of Bioinformatics and Structural Biology, National Tsing Hua University, Hsinchu, 30043 Taiwan; 3 Department of Biotechnology and Bioindustry Sciences, National Cheng Kung University, Tainan, 701 Taiwan; 4 Department of Physics, National Tsing Hua University, Hsinchu, 30043 Taiwan; 5 Institute for Protein Research, Osaka University, Suita, Osaka, 565-0871 Japan; 6 Institute of Chemistry, Academia Sinica, Nankang, Taipei, 11529 Taiwan; 7 Noyes Laboratory 127-72, California Institute of Technology, Pasadena, CA 91125 USA

## Abstract

The membrane-embedded quinol:fumarate reductase (QFR) in anaerobic bacteria catalyzes the reduction of fumarate to succinate by quinol in the anaerobic respiratory chain. The electron/proton-transfer pathways in QFRs remain controversial. Here we report the crystal structure of QFR from the anaerobic sulphate-reducing bacterium *Desulfovibrio gigas* (*D*. *gigas*) at 3.6 Å resolution. The structure of the *D*. *gigas* QFR is a homo-dimer, each protomer comprising two hydrophilic subunits, A and B, and one transmembrane subunit C, together with six redox cofactors including two *b*-hemes. One menaquinone molecule is bound near heme *b*_L_ in the hydrophobic subunit C. This location of the menaquinone-binding site differs from the menaquinol-binding cavity proposed previously for QFR from *Wolinella succinogenes*. The observed bound menaquinone might serve as an additional redox cofactor to mediate the proton-coupled electron transport across the membrane. Armed with these structural insights, we propose electron/proton-transfer pathways in the quinol reduction of fumarate to succinate in the *D*. *gigas* QFR.

## Introduction

*Desufovibrio gigas* (*D*. *gigas*), an anaerobic sulphate-reducing bacteria (SRB) with versatile anaerobic respiratory mechanisms^1^, requires specific enzymes to mediate the anaerobic respiratory processes that catalyze the sequential reduction reactions to obtain energy. The terminal electron acceptors in these reactions are moderate oxidants, such as sulphate, sulphite, other sulphur compounds, and fumarate, rather than the strong oxidants, e.g. dioxygen, utilized in aerobic respiration. One of these crucial enzymes is quinol:fumarate reductase (QFR), which is an integral membrane protein with three subunits: a flavoprotein (subunit A), an iron-sulphur protein (subunit B), and a membrane-embedded subunit (subunit C). QFR catalyzes the coupled reduction of fumarate to succinate with the oxidation of hydroquinone (quinol) to quinone on opposite sides of the inner cytoplasmic membrane. The reverse reaction, namely, the coupled oxidation of succinate to fumarate with the reduction of quinone to quinol, is catalyzed by the well-studied succinate:quinone reductase (SQR), often referred to as complex II^2^ in the respiratory electron-transport chain of aerobic organisms^3,4^.

In this study, we report on the X-ray crystal structure of QFR from *D*. *gigas*. Two prokaryotic QFR structures have already been described: ones from *Wollinella succinogenes* (*W*. *succinogenes* – PDB ID 1QLB and 2BS2)^5,6^ and *Escherichia coli* (*E*. *coli* – PDB ID 1L0V)^7^. In addition, one eukaryotic mitochondrial QFR from *Ascaris suum* (*A*. *suum* – PDB ID 3VR8)^8^ has also been determined. Structurally, these QFRs all contain two hydrophilic subunits, namely, the flavoprotein and the iron-sulphur protein. However, two hydrophobic membrane-embedded subunits are associated with the *E*. *coli* and *A*. *suum* QFRs, whereas only one membrane-associated subunit is seen in the *W*. *succinogenes* enzyme. As anticipated, the structures of the hydrophilic subunits are similar among these QFRs, but major structural variations are found in the membrane-embedded subunits^9^. In addition, the three QFRs reveal distinct structural arrangements of the redox cofactors: FAD-[2Fe:2S]-[4Fe:4S]-[3Fe:4S]-heme *b*_H_-heme *b*_L_ in *W*. *succinogenes*; FAD-[2Fe:2S]-[4Fe:4S]-[3Fe:4S]-Q_P_-Q_D_ in *E*. *coli* (Q_P_: the proximal menaquinone, Q_D_: the distal menaquinone); and FAD-[2Fe:2S]-[4Fe:4S]-[3Fe:4S]-heme *b* in *A*. *suum*. Q_D_ is apparently not redox-active in *E*. *coli* QFR^10,11^. Based on these structural findings, electron transfer mechanisms have been proposed to account for the quinol reduction of fumarate to succinate to enable the anaerobic microorganisms to grow on fumarate^4,5,7^. Two protons are consumed in the two-electron reduction of the fumarate, and it has been proposed that the transfer of these protons to the cytoplasm is coupled to the electron transfers, the so-called E pathway^12^, with key residues identified or proposed in the subunit C of *W*. *succinogenes*^13,14^.

To clarify the mechanism of QFR, we have cultured large amounts of *D*. *gigas* cells under anaerobic conditions and purified the QFR directly from the cell membranes for structural studies. We have determined the crystal structure of QFR from *D*. *gigas* at 3.6 Å resolution by X-ray crystallography. All the redox cofactors and a bound menaquinone are revealed in our structure. The menaquinone-binding site is different from that proposed for the QFR structure of *W*. *succinogenes*. Detailed comparison of the structures and redox cofactors among the various QFRs has allowed us to propose the pathways of electron and proton transfers in QFR from *D*. *gigas* during turnover.

## Results

### Overall structural fold

**Figure 1:**
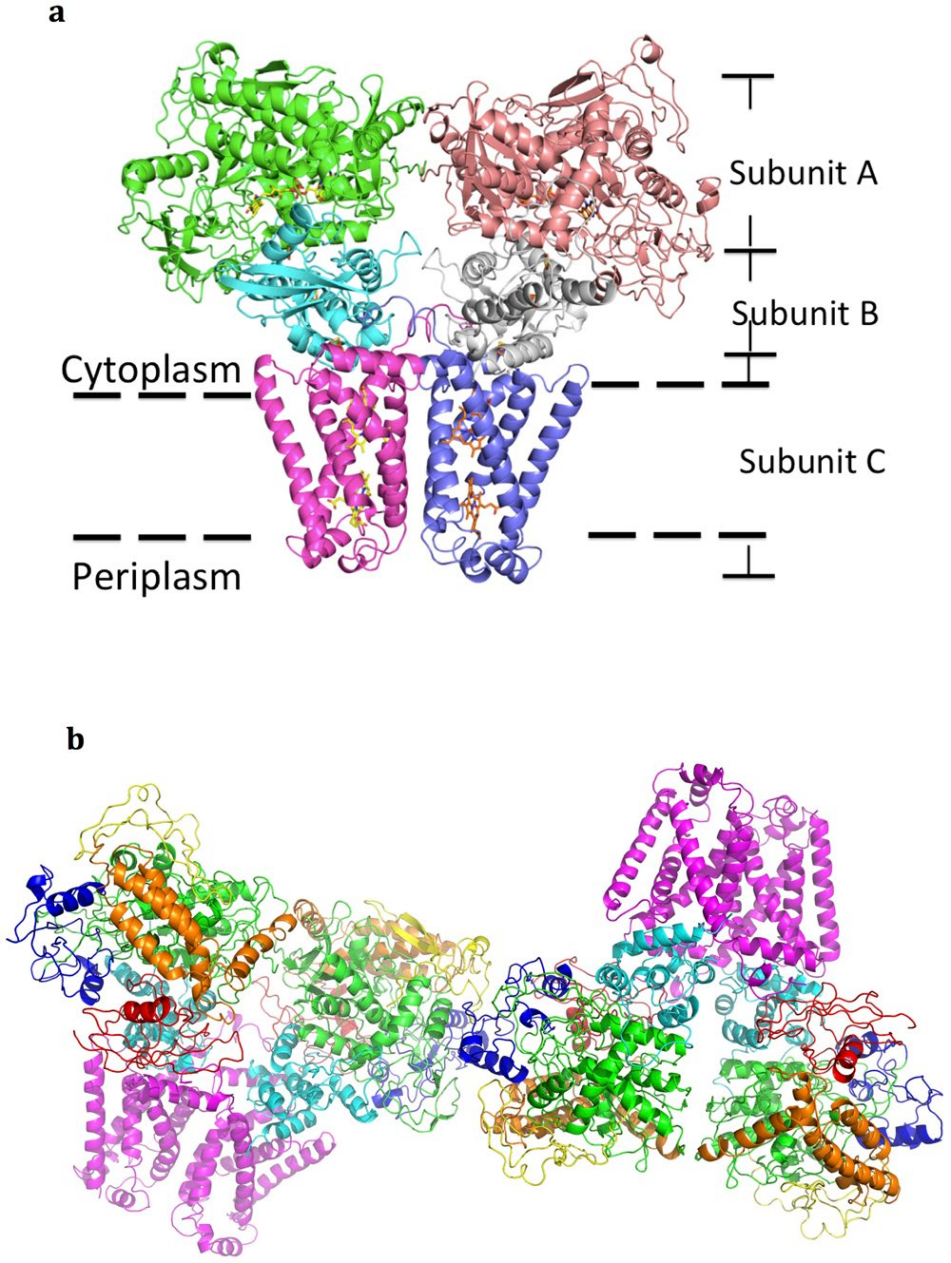
The overall architecture of dimeric QFR from *D. gigas*. (**a**) The homo-dimer formed by two complexes of the subunits A (shown in green and brown), B (cyan and gray) and C (pink and purple) on the inner membrane. The redox cofactors, hemes, iron-sulphur clusters and FAD are shown by sticks. (**b**) Two homo-dimers formed by four sets of subunits A, B and C in the asymmetric unit. The FAD-binding domain (green), the capping domain (red), the helical domain (orange) and the C-terminal domain (yellow) of the subunit A are shown, respectively. The N-terminal plant-ferredoxin domain (red) and a C-terminal bacterial-ferredoxin domain (cyan) of the subunit B and the whole C subunit (pink) are shown.

Two hetero-trimeric complexes, each comprising subunits A, B and C, form one stable homo-dimer (A_2_B_2_C_2_) with major contacts between two C subunits (Fig. 1a). There are two homo-dimers 2(A_2_B_2_C_2_) of the hetero-trimeric complex (ABC) in one asymmetric unit (Fig. 1b), but the contact interface is insufficient to form a stable tetramer in the crystal packing, with averaged buried surface area of only ~382 Å^2^ between only one subunit A from each of the two homo-dimers (chains A and I) (Fig. S1), consistent with our observation that the protein elutes as a homo-dimer in size-exclusion chromatography during protein purification. The two membrane-spanning domains of the two homo-dimers 2(A_2_B_2_C_2_) in the crystal are oriented in opposite directions. Since this could not be the proper scenario for QFR within the cell membrane, the homo-dimer (A_2_B_2_C_2_) must be the biological functional assembly.

Within each homo-dimer, (A_2_B_2_C_2_), the averaged buried areas are ~471 Å^2^ between the two A subunits, negligible between the two B subunits, and 1340 Å^2^ between the two C subunits; the portion of the buried surfaces of the two A subunits and the two C subunits are 2.1 and 9.5%, respectively. The main driving force in the homo-dimer formation thus comes from interactions between the C subunits. In support, the complex formation significance score (CSS) of the two C subunits and the two A subunits calculated by PISA^15^ (http://www.ebi.ac.uk/pdbe/prot_int/pistart.html) are ~0.22 and ~0.02 (Fig. S2), respectively, indicating that the formation of the homo-dimer, (A_2_B_2_C_2_), arises from contact of the two C subunits: the CSS value increases from 0 to 1 as the interface relevant to complex formation increases. The interactions between the two C subunits contain three important hydrogen bonds, two from Arg85-Gln91 and one from Gly151-Thr135 (Fig. S2).

### Subunit A (FAD-binding protein)

The flavin adenine dinucleotide (FAD)-binding protein (subunit A) of *D*. *gigas* QFR is composed by a total of 627 residues, with a FAD-binding domain (A1–260 and A366–437), a capping domain (A261–360), a helical domain (A438–555), and the C-terminal domain (A556–622) containing an antiparallel β-sheet (A564–569 and A578–583). Without interpretable electron density, the structure of the C-terminus A623–627 is too flexible to be built. This structure of subunit A differs from that of *W*. *succinogenes* QFR in that the latter contains two additional C-terminal helices, one long and one short.

The domain architecture of subunit A of *D*. *gigas* is similar to those of *W*. *succinogenes*, *E*. *coli* and *A*. *suum*. The location of FAD within the FAD-binding domain is similar for all four QFRs. The C8M methyl group of the flavin of FAD binds to the FAD-binding domain through a covalent bond with residue His-A43, which is conserved among these A subunits of QFRs. A fumarate molecule is also observed near the FAD in the A subunit. This position of the fumarate is conserved between *D*. *gigas* and *W*. *succinogenes* QFRs as well.

During the purification, there was a contamination of a ~60-kD protein after several types of columns (Fig. S3a). The ~60-kDa protein, accounting for about 30% of total proteins, was identified as the subunit A of QFR, lacking the N-terminal fragment, according to analysis by the liquid chromatography-tandem mass spectrometry (LC/MS/MS) (data not shown). The ~60-kDa protein might be a proteolytic fragment of the subunit A even though a protease inhibitor (cOmplete^TM^ EDTA-free protease inhibitor cocktail, Roche) was added in all processes of cell disruption and protein purification. However, we cannot rule out the possibility that there is another isoform of the subunit A of *D*. *gigas* QFR without the N-terminal fragment.

### Subunit B (iron-sulphur protein)

The iron-sulphur protein of *D*. *gigas* QFR comprises a total of 264 residues, which can be partitioned into an N-terminal plant-ferredoxin domain (B1–106) and a C-terminal bacterial-ferredoxin domain (B107–240). The C-terminal bacterial-ferredoxin domain contains the [3Fe:4S] cluster coordinated by Cys-B156, Cys-B161 and Cys-B208, as well as the [4Fe:4S] cluster coordinated by Cys-B151, Cys-B154, Cys-B157 and Cys-B218. This domain is in contact with the hydrophobic subunit C, and the [3Fe:4S] and [4Fe:4S] clusters are located near the menaquinone site in subunit C (*vide infra*). The N-terminal plant-ferredoxin domain is in contact with the hydrophilic subunit A, and it contains only one [2Fe:2S] cluster coordinated by Cys-B57, Cys-B62, Cys-B65 and Cys-B77. This [2Fe:2S] cluster is located near the fumarate-reducing site, and presumably participates in shuttling the electrons from the [3Fe:4S] and [4Fe:4S] clusters of subunit B downstream to the FAD in subunit A for the ultimate hydride transfer from the reduced FAD, or FADH, to the fumarate substrate. All the coordinating cysteine residues to the iron sulphur clusters are structurally conserved in the B subunits of *D*. *giga*s, *W*. *succinogenes*, *E*. *coli* and *A*. *suum*. The spatial arrangements of the Fe-S clusters in *D*. *gigas* are also similar to those in *W*. *succinogenes*, *E*. *coli* and *A*. *suum*. Thus, the electron transfer activities mediated by the Fe-S clusters within the subunit B should be similar among the four QFRs.

### Subunit C (membrane-embedded protein)

This membrane-embedded subunit C of *D*. *gigas* QFR comprises a total of 218 residues organized into mainly seven helices, including five transmembrane helices: α1 (C16–41), α3 (C69–83), α5 (C107–134), α6 (C156–173) and α7 (C183–211); one periplasmic helix α2 (C49–56); and one cytoplasmic helix α4 (C91–104). Two *b*-type hemes are associated with the helix-bundle. The high-potential heme *b*_H_ is proximal to the hydrophilic A and B subunits, and has as axial ligands His-C79 and His-C166 on helices α3 and α6, respectively. The low-potential heme *b*_L_, which is located distal to the hydrophilic A and B subunits, is coordinated to His-C38 and His-C129 on helices α1 and α5, respectively. These four helices, α1, α3, α5 and α6, which coordinate the two hemes, form the so-called the four-helix motif that exists in other di-heme proteins^16,17^. These four histidine residues are conserved in QFRs of *D*. *gigas* and *W*. *succinogenes*. They are missing for the enzyme in *E*. *coli* (Fig. S4), and only two are found in *A*. *suum* QFR. Consistent with this primary sequence information, there is no bound heme seen in the membrane-embedded subunit C of *E*. *coli*^7^ and only one heme is associated with the *A*. *suum* enzyme^8^.

Of the remaining α-helices, helix α4 interacts with the iron-sulphur protein (subunit B) at the top near the cytoplasm, and helix α2 is situated at the bottom near the periplasm; helix α7 is also a transmembrane segment, which, together with the four-helix motif— α1, α3, α5 and α6, forms the transmembrane helix bundle sequestered within the inner membrane.

### Bound detergents in *D. gigas* QFR

One *n*-dodecyl β-D-maltoside (DDM) molecule from the detergent used to solubilize and stabilize the protein is found in subunit C of *D*. *gigas* QFR (Fig. S5a). The polar head group of the DDM forms a hydrogen bond with Lys-C95 of QFR; the hydrophobic tail of the DDM interacts with a hydrophobic pocket formed by the hydrophobic residues Ile-C81, Pro-C88, Phe-C89, Trp-C94, Val-C116, Ala-C119 and Ile-C120 (Fig. S5b). A second DDM molecule is bound at the same location in the other subunit C of the homo-dimer. These bound DDM molecules are considered to mimic membrane lipids, indicating that QFR of *D*. *gigas* might utilize residue Lys-C95 to interact with the polar head-group in the inner leaflet of the lipid bilayer, and the tail of the lipid molecule might be sequestered by the hydrophobic pocket within the subunit C of QFR.

### Structural comparison of QFRs

**Figure 2:**
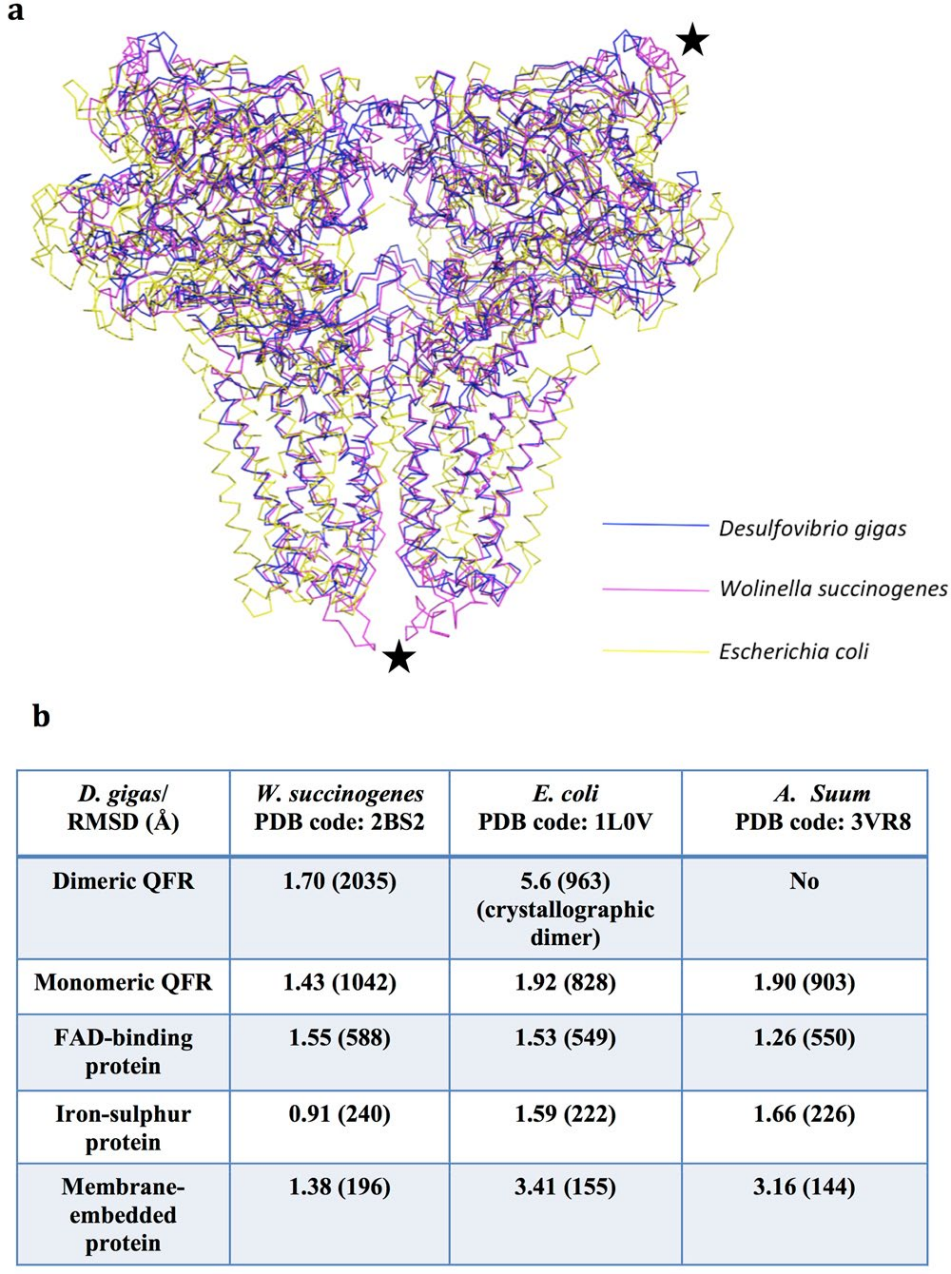
Structural comparisons of QFRs from *D*. *gigas*, *W*. *succinogenes*, *E*. *coli*. and *A*. *suum*. (**a**) Superimposed structures of dimeric QFRs from *D*. *gigas*, *W*. *succinogenes* and *E*. *coli*. Dimeric QFRs from *D*. *gigas*, *W*. *succinogenes* and *E*. *coli* (from crystallographic symmetry) are colored blue, magenta and yellow, respectively. The major structurally variable areas between *D*. *gigas and W*. *succinogenes* QFRs are labeled as black stars. (**b**) RMSDs between *D*. *gigas* QFR and *W*. *succinogenes* QFR, *E*. *coli* QFR as well as *A*. *suum* QFR are listed. The parentheses represent the numbers of residues aligned using *SSM* in *Coot*.

Superimposed structures of the dimeric QFRs, monomeric QFRs, individual subunits A, B and C from *D*. *gigas* with those of the prokaryotic *W*. *succinogenes* (PDB code: 2BS2) and *E*. *coli* (as a crystallographic dimer, PDB code: 1L0V) show root-mean-square-deviations (RMSD) 1.7/5.6, 1.43/1.92, 1.55/1.53, 0.91/1.59 and 1.38/3.41 Å (Fig. 2b), respectively, using the secondary-structure matching (*SSM*)^18^ tool in *Coot*^19^. Since the eukaryotic QFR structure from *A*. *suum* exists as a monomer in solution and does not exhibit the similar dimeric arrangement as *D*. *gigas* QFR in the crystal packing (Fig. S6), only monomer structures between *D*. *gigas* and *A*. *suum* QFRs (PDB code: 3VR8) are compared: the RMSDs calculated by *SSM* are 1.90, 1.26, 1.66 and 3.16 Å for monomeric QFR, subunits A, B, and the membrane-embedded subunit C, respectively (Fig. S6). From these comparisons, it is evident that the hydrophilic subunits (subunits A and B) of *D*. *gigas*, *W*. *succinogenes*, *E*. *coli* and *A*. *suum* are relatively similar, whereas the hydrophobic subunit(s) (subunit C) exhibit major structural variations (Figs 2 and S6).

As shown in Fig. 2a, superimposition of the three prokaryotic dimeric QFR structures shows that there are minor molecular contacts between the two subunits A of the homodimer from *D*. *gigas* and *W*. *succinogenes*, but no molecular contact between the two subunits A from *E*. *coli* QFR. Moreover, low CSS (~0.05), but high CSS (~0.29) and (~0.22), are shown between the two membrane-embedded subunits C of *E*. *coli* (as a crystallographic dimer), *W*. *succinogenes* and *D*. *gigas* QFRs, respectively. These observations are consistent with the dimeric form (A_2_B_2_C_2_) of QFRs of *D*. *gigas* and *W*. *succinogenes* and the monomeric form (ABC) of QFR of *E*. *coli* in solution^7,20,21^.

The structural variations between *D*. *gigas* and *W*. *succinogenes* QFR are confined mainly to the C-terminal regions of subunits A and a loop between helices 2 and 3 of subunits C (Fig. 2a). There is a pair of long and short helices at the C-terminal region of subunit A in QFR from *W*. *succinogenes*; this helix pair is absent in QFR from *D*. *gigas* as well as from *E*. *coli*. The biological function of this pair of helices in QFR from *W*. *succinogenes* is unclear. As for the loop between the helices 2 and 3 in the C subunits, QFR from *W*. *succinogenes* contains a longer loop with eight extra residues, compared with QFRs of *D*. *gigas* and *E*. *coli*. The longer loop with a proposed critical residue Glu-C66 has been implicated in the binding of the menaquinol substrate in QFR from *W*. *succinogenes*^13^ for the fumarate reduction. Without this long loop, as in the case of QFR from *D*. *gigas*, menaquinol binding might be affected here. The structural variation in this region might imply that the menaquinol-binding mode of QFR from *D*. *gigas* is different from that of *W*. *succinogenes*.

### The deformed cavity for the head-group binding of the proposed menaquinol substrate

**Figure 3:**
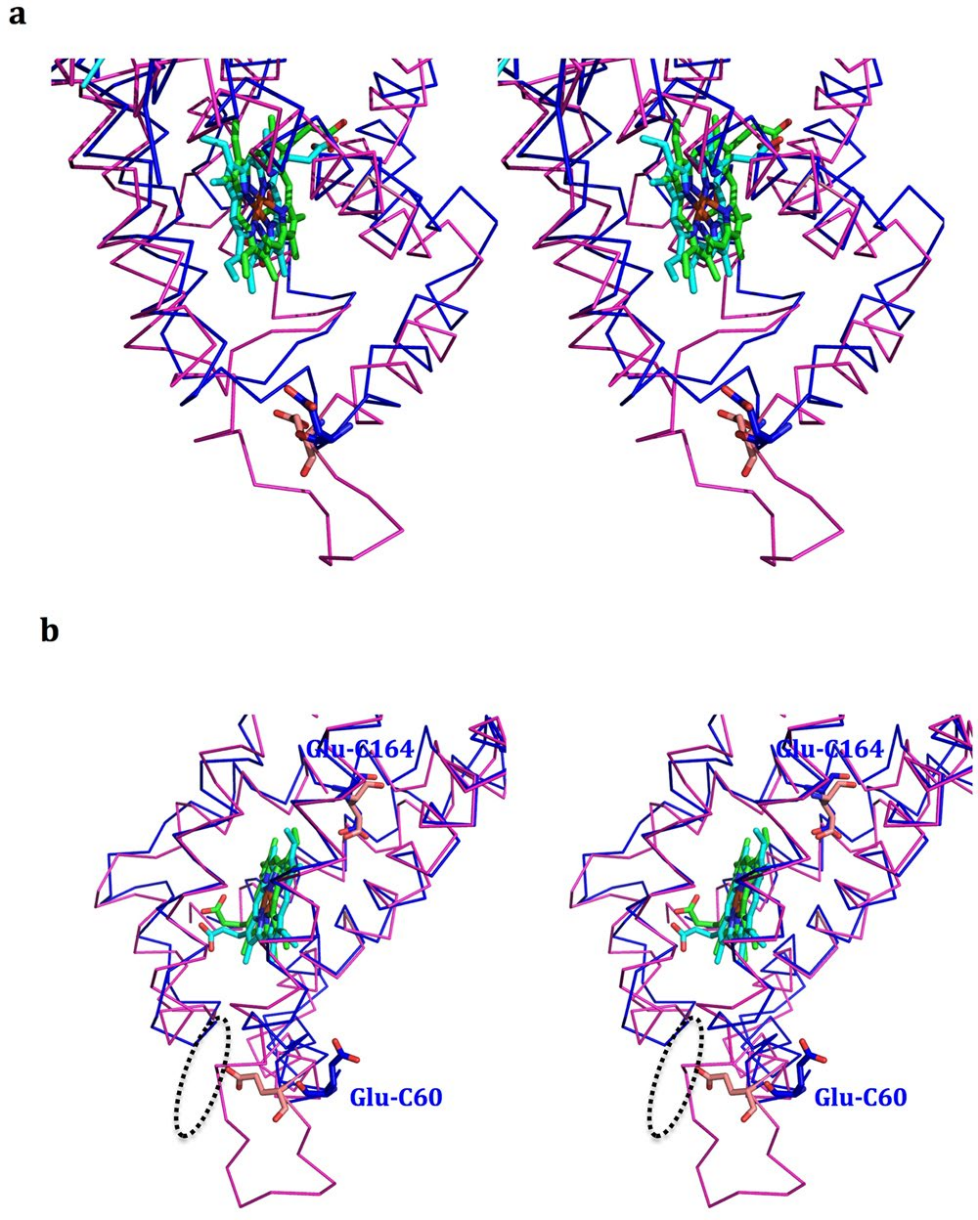
Superimposed structures of the previously proposed menaquinol-binding cavities of *W*. *succinogenes* and *D*. *gigas* QFR. (**a**) A front stereo view (**b**) a side stereo view of the superimposed structures of the subunits C containing the previously proposed menaquinol-binding cavities from *W*. *succinogenes* and the similar region of *D*. *gigas* are shown in magenta and blue ribbon, respectively. The *b*_L_ hemes of *D*. *gigas* and *W*. *succinogenes* are shown as green and cyan sticks, respectively. The key residues Glu-C66 in *W*. *succinogenes* QFR and Glu-C60 in *D*. *gigas* QFR with distinct conformations are shown as light magenta and blue sticks, respectively, whereas the other key residues Glu-C180 in *W*. *succinogenes* QFR and Glu-C164 in *D*. *gigas* QFR with the similar conformations are also shown as light magenta and blue sticks, respectively. The proposed menaquinol-binding cavity in *W*. *succinogenes* QFR is presented in a dashed elliptical circle.

In an earlier study, individual replacements of Glu-C66 and Glu-C180 with Gln by site-directed mutagenesis of *W*. *succinogenes* QFR showed that both of these bacterial mutants could no longer grow under fumarate^13,14^. These results have implicated Glu-C66 and Glu-C180 of *W*. *succinogenes* QFR in the proton transfers that must accompany the transport of the electrons across the membrane during the reduction of fumarate by the quinol substrate. As shown in Fig. 3a and b, Glu-C66 is on the loop between helices 2 and 3 of QFR from *W*. *succinogenes*. Glu-C66 and a proposed cavity near the loop were suggested to be involved in the binding of the head group of the menaquinol substrate. In this region, there are actually two proposed menaquinol-binding cavities: one is for the head group, and the other, close to the *b*_L_ heme, is for the hydrophobic tail of the menaquinol^13^.

In *D*. *gigas* QFR, however, Glu-C60 (the structural analog of Glu-C66 in *W*. *succinogenes* QFR) adapts a structural orientation such that the side chain points away from the proposed menaquinol-binding cavity in *W*. *succinogenes* QFR (Fig. 3a and b). In addition, the loop containing Glu-C60 is shorter by eight residues (Fig. S4). As a result, the corresponding cavity in *D*. *gigas* QFR is deformed so that it can no longer accommodate the head group of a menaquinol substrate (Fig. 3). These observations, the different orientation of the Glu-C60 and the deformed cavity for the head group, might be taken to infer that *D*. *gigas* QFR possesses a different menaquinol-binding mode, wherein the menaquinol substrate molecule binds more closely to the *b*_L_ heme to take advantage of the more hydrophilic cavity near this heme in *D*. *gigas* QFR compared with that in *W*. *succinogen*es QFR.

However, Glu-C164 in *D*. *gigas* QFR, analog of the other essential residue Glu-C180 in *W*. *succinogenes* QFR, exhibits the similar structural conformation as in *W*. *succinogenes* QFR (Fig. 3b), indicating that Glu-C164 might play a similar crucial role in proton transfer(s) in *D*. *gigas* QFR. Other important residues, which might be involved in the electron-transfer and proton-transfer pathways, are discussed in the following sections.

### Bound menaquinone near the low-potential heme (*b*_L_)

The menaquinol-oxidation site in *D*. *gigas* QFR (henceforth referred to as MQ1) is not known. To explore this issue, we search for potential menaquinol-binding positions based on the electron density maps (SIGMAA-weighted *F*_o_ − *F*_c_ and 2*F*_o_ − *F*_c_). The deformed cavity near Glu-C60 in *D*. *gigas* QFR, the binding cavity previously proposed in *W*. *succinogenes*, shows no extra density for a menaquinol molecule, in support of a menaquinol-binding location elsewhere on the periplasmic side of subunit C in *D*. *gigas* QFR. In any case, we do not expect menaquinol to be bound to the enzyme at the MQ1 site when fumarate is associated with the QFR, as the substrate will be readily oxidized. Neither should menaquinone, the product of the substrate oxidation, be seen in the crystal structure. The quinone should be only weakly associated with the QFR during catalytic turnover of the enzyme. The concentration of menaquinone in the cells is very low under growth conditions in any case.

**Figure 4:**
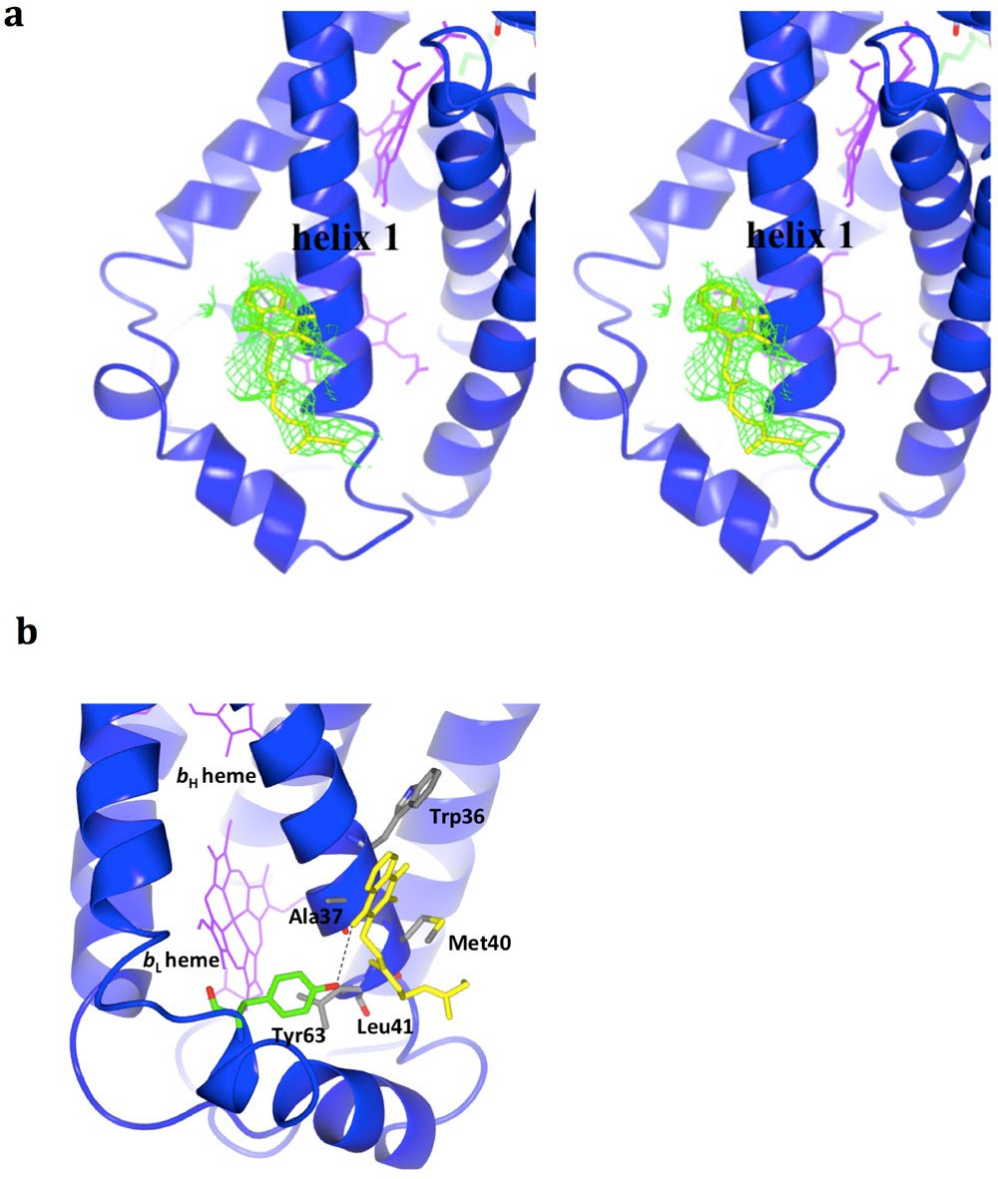
The bound menaquinone (MK) at the MQ2 site near the *b*_*L*_ heme in *D*. *gigas* QFR. (**a**) A stereo view of the bound MK with the electron density. The SIGMAA weighted *F*_o_ − *F*_c_ density maps are shown as green mesh at σ 1.2. MK and hemes are shown as yellow and magenta sticks, respectively, in subunit C as cartoon helices. (**b**) The hydrophobic pocket is formed with Trp36, Ala37, Met40 and Leu41, which are colored grey. Tyr63 (green stick) of subunit C forms a hydrogen bond (dashed line) with MK (yellow stick). Hemes are shown as magenta sticks.

Nevertheless, we have identified a menaquinol or menaquinone molecule with the appropriate electron density near helix 1 (C-helix1) of one subunit C among the four subunit C molecules in the asymmetric unit (Fig. 4a). We have designated this location in the *D*. *gigas* QFR as the MQ2 site, to distinguish this site from the MQ1 site, where substrate oxidation presumably occurs. At this site, the hydrocarbon tail of the menaquinol or menaquinone molecule is sequestered within a hydrophobic pocket formed by Met-C40, Leu-C41, Trp-C36 and Ala-C37 on C-helix1; and the headgroup is directly bound to Tyr-C63 on another short fragment *via* a hydrogen bond (Fig. 4b). With the limitation of X-ray crystallography, we could not distinguish between a quinol and a quinone occupying the MQ2 site. However, as a “cofactor”, it is most certainly oxidized on the basis of chemical considerations, and we henceforth refer to it as a menaquinone (MK). According to the electron density, only the electron densities of two isoprenoid units of this MK were observed. Notably, we also tried to fit a DDM molecule, which was used for crystallization, into this extra electron density. However, the geometry of the head group of DDM could not be fitted appropriately based on the electron density.

**Figure 5:**
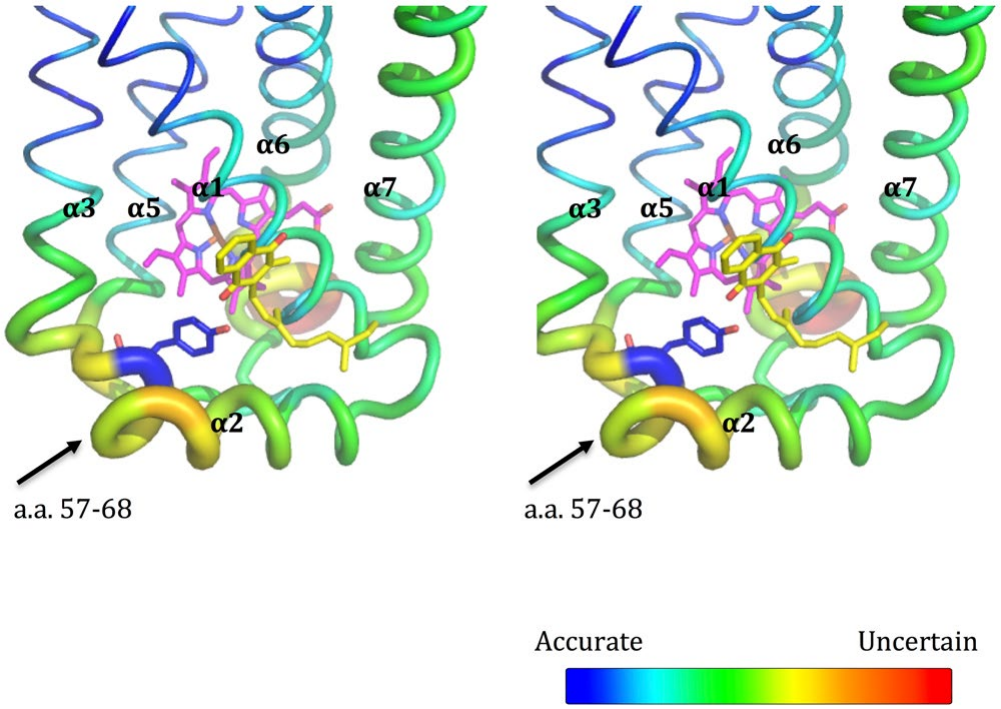
*B*-factors and structural flexibility of subunit C. A stereo view of the *B*-factor variations of subunit C (C-chain) is presented. The heme is shown as magenta sticks. The bound menaquinol molecule (MK) and Tyr-C63 are shown as yellow and blue sticks, respectively. The flexible region (a.a. 57–68) between helices 2 and 3 is indicated with a black arrow.

Evidently, this MK is readily lost during protein purification as only one MK molecule is discerned per asymmetric unit of the protein crystal. The conformation of this short fragment (a.a. 57–68) containing the Tyr-C63, which is located between helices 2 and 3, is highly variable in the crystal structure of *D*. *gigas* QFR (Fig. 5). The four short fragments of the two dimeric QFR in the asymmetric unit exhibit conformations more variable than other regions, indicating that their dynamic property or flexibility might be related to the binding of menaquinones (Fig. S7). The Tyr-C63 that binds MK exhibits the lowest *B*-factor compared to the three other free Tyr63 residues of the asymmetric subunits C (i.e., G-chain, K-chain and O-chain), which might result from the formation of the hydrogen bond between the side-chain O–H of the C-chain Tyr-C63 and the bound MK. Interestingly, this Tyr-C63 residue, which is specific to *D*. *gigas* (Fig. S4), is positioned near residue Glu-C60, the analog of Glu-C66 in *W*. *succinogenes* QFR that has been implicated in the proton transfers accompanying the electron transport during the catalytic turnover. Finally, there is a highly variable region (a.a. C147-C152) behind the *b*_L_ heme, but the dynamic role of this region is unclear (Fig. 5).

**Figure 6:**
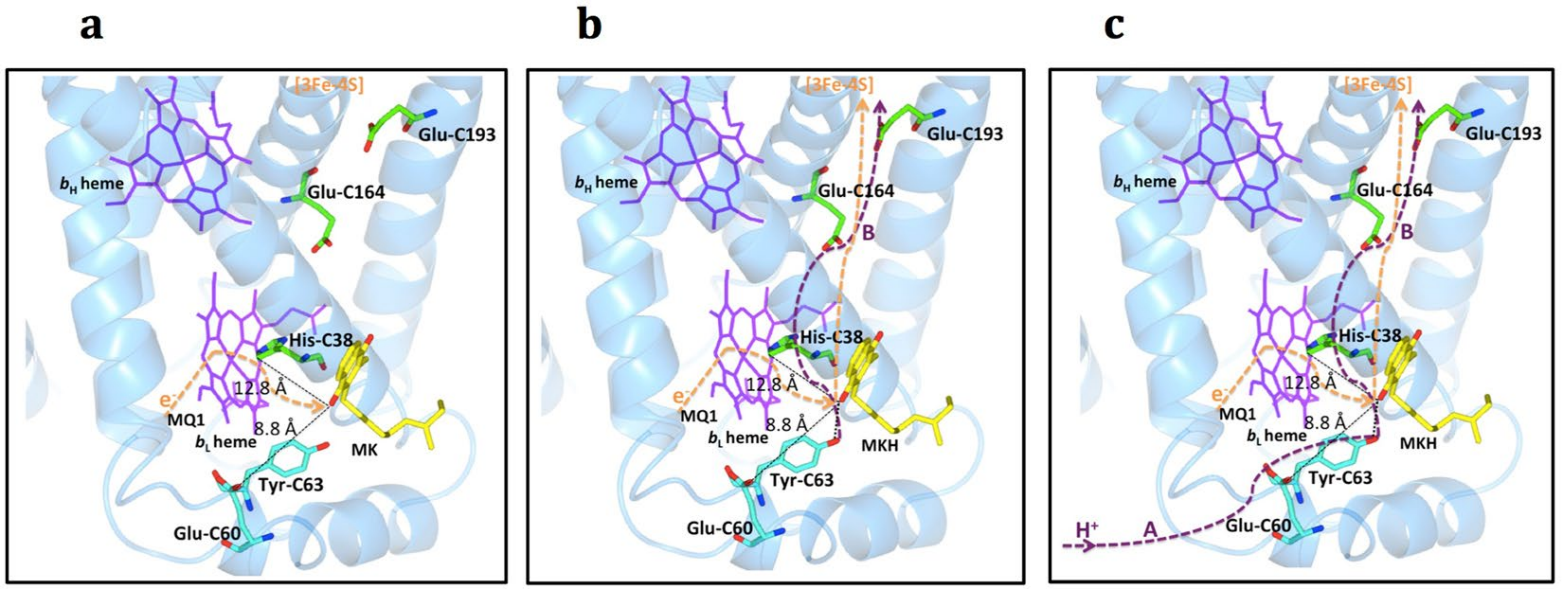
Possible sequential synchronous movement of the proton in concert with the electron flow. (**a**) The initial electron flow from the reduced menaquinol (MKH_2_) at the MQ1 site to the MK at the MQ2 site. (**b**) The following synchronous proton movement in concert with the electron flow to the [3Fe:4S] are shown. (**c**) The complete electron/proton pathways from periplasm to cytoplasm are shown. The bound MK or MKH at the MQ2 site are shown as yellow sticks and the subunit C of QFR from *D*. *gigas* is shown in light blue. Two potential proton acceptors near the MQ2 site, His-C38, and Glu-C60 are shown as green and cyan sticks, respectively. In addition, Tyr-C63 also colored cyan on the potential proton-transfer pathway from periplasm could form a hydrogen bond with the bonund MKH during the proton transfer. The distances between MKH and His38, as well as between MKH and Glu-C60, are labeled in black. The possible proton pathway and the proton-coupled electron transfer pathway are shown as purple and orange dotted lines, respectively. The hydrogen bond between the MKH and the Tyr-C63 is shown as black dotted lines. Hemes are shown as magenta sticks.

Moreover, this MQ2 site, or the bound MK, is located near (~13 Å) the low-potential *b*_L_ heme (Fig. 5). This observation, together with the conformational flexibility of the short fragment (a.a. 57–68) containing the Tyr-C63 described earlier, has led us to surmise that MK might serve as an electron carrier between heme *b*_L_ and [3Fe:4S] in the electron-transfer pathway of *D*. *gigas*. In other words, MK could be gating the proton-coupled-electron transfer from heme *b*_L_ to [3Fe:4S] (Fig. 6a). Reduction of MK gives the semi-menaquinone anion (MKH), which should stabilize the hydrogen bonding of the Tyr-C63 with the cofactor and lower the pK_A_ of this tyrosine further. Upon reoxidation, as the electron is transferred from the MKH downstream to the [3Fe:4S], electrostatic interaction between the electron and the proton of the Tyr-C63 hydrogen-bond provides a linkage to drive the synchronous movement of the proton across the membrane in concert with the electron flow (Fig. 6b). In this manner, proton-coupled electron transfer across the membrane is achieved (Fig. 6c).

### Electron-transfer pathway(s)

**Figure 7:**
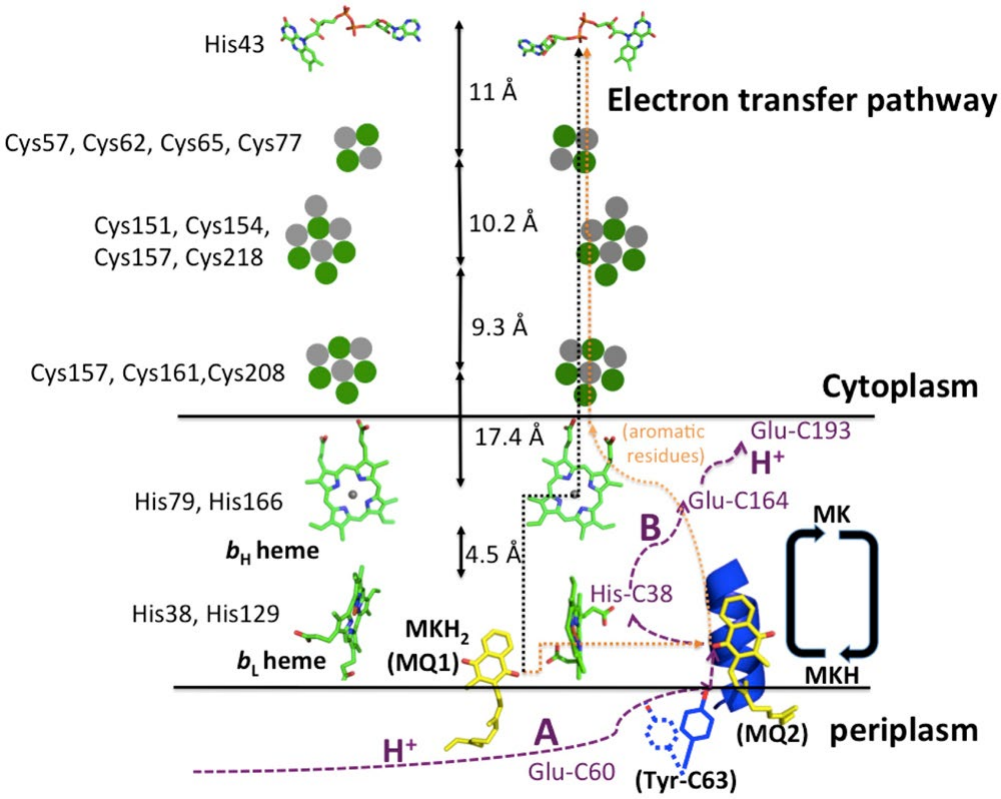
Proposed electron/proton-transfer pathways in QFR of *D*. *gigas*. The fully reduced manaquinol (MKH_2_ at the MQ1 site) and the oxidized menaquinone (at the MQ2 site) are shown as yellow sticks. The fully oxidized and semi-oxidized menaquinones are labeled as MK and MKH, respectively. The MK and MKH, newly identified in this work, are bound at the hydrophobic pocket on the C-helix1 (in green). The proposed bifurcation E pathways are labeled with black and orange dot lines and the potential flow pathways of protons generated in the oxidation of MKH_2_ are labeled as purple dotted lines and classified as proton pathways A and B. The redox cofactors, FAD (upper sticks), hemes (bottom sticks) and iron-sulphur clusters (gray and green balls), are shown on the electron transfer pathway. The residues ligating the redox cofactors are labeled black and the proposed proton acceptors are labeled purple. OM and IM stand for the bacteria outer and inner membranes, respectively. Two black lines indicate the inner membrane. The movement of Tyr-C63 is also shown after bound with the MKH with a hydrogen bond.

Based on the arrangement of the redox cofactors in the crystal structure, including the bound MK at the MQ2 site, as well as their redox potentials, it is possible to postulate the E-pathway(s) in QFR of *D*. *gigas* (Fig. 7). First, we propose that, upon oxidation of the menaquinol at the MQ1 site, there is bifurcation of the electron flow from the substrate to the high-potential heme *b*_H_ and the low-potential heme *b*_L_. Second, we suggest that heme *b*_L_ serves as a relay station to transfer one of these electrons to the [3Fe:4S] center via the MK at the MQ2 site, and from the [3Fe:4S] site, the electron passes on to the [4Fe:4S]-[2Fe:2S]-FAD, as mentioned earlier. To replenish the proton of the Tyr-C63 side chain after the proton-coupled electron transfer across the membrane, a proton must be taken up from the periplasm and transported *via* proton acceptors located within the periplasmic side of subunit C to the side chain of Tyr-C63 as it is known that the hydroxyl group of tyrosine can serve to transfer protons^22^. Potential proton acceptors might include Glu-C60, among others. Similarly, there must be proton acceptors within the transmembrane domain toward the cytoplasmic side to relay the proton that has been translocated across the membrane toward the cytoplasm. Potential proton acceptors here include residues Glu-C164 and Glu-C193 (Fig. 6c), and possibly other acidic residues Asp-C108, Asp-C21 and Glu-C93, which are at or near the cytoplasmic helix α4 (C91–104).

As to the other reducing equivalent, the one transferred to the *b*_H_ heme in the bifurcation of the electron flow from the menaquinol substrate mentioned earlier, we propose a direct transfer of this electron from heme *b*_H_ to the [3Fe:4S] (Fig. 7). The high-potential heme *b*_H_ is in relatively close proximity to the hydrophilic A and B subunits. The redox potentials of heme *b*_H_ (0 mV), heme *b*_L_ (−150 mV) and MK (−67 to −74 mV) near pH ~7 are also suggestive of such a possible scenario^23,24^. However, for a proton-coupled electron transfer event to take place between subunit C and subunit B, there will have to be redox linkage between heme *b*_H_ and the pK_A_ of an amino acid residue within subunit C, as well as conformational gating of a proton transfer across the membrane in concert with the electron transfer. Possible proton donors might include His-C38, which is associated with heme *b*_L_. The *b*_H_ and *b*_L_ hemes in *D*. *gigas* QFR are in sufficient close contact (only ~4.5 Å apart) that we can expect a redox interaction between them.

At this juncture, however, we cannot rule out the possibility of also transferring the electron from the *b*_H_ heme *via* heme *b*_L_ to MK at the MQ2 site for the proton-coupled electron transfer to the [3Fe:4S] center, as we have described earlier for the other reducing equivalent. Indeed, the two hemes are in sufficiently close juxtaposition that the electrons can redistribute readily between these iron porphyrins, provided that their operating redox potentials and their relative orientation, are favorable. Examination of the crystal structure reveals that the two hemes are closely perpendicular to each other, a geometry that is known to be unfavorable toward facile inter-heme electron transfers^25^. Thus, on the basis of this structural information, as well as the relative redox potentials between heme *b*_H_ and *b*_L_ (150 mV)^23^, we are inclined to favor different electron transfer pathways for the two reducing equivalents of the menaquinol substrate during the catalytic turnover of *D*. *gigas* QFR.

## Discussion

Upon oxidation of menaquinol in QFRs, two electrons are transferred across the membrane from the periplasm to the cytoplasm. QFRs usually comprise zero to two hemes in the membrane-embedded subunit, but the role of these hemes has not been fully characterized in the E pathway of QFRs^26^. The two-electron transfer through hemes and Fe-S clusters was previously proposed based on the locations of the redox cofactors in the QFR structure of *W*. *succinogenes*^5^. Native SQR of *E*. *coli* contains one heme; however, an *E*. *coli* SQR mutant lacking this heme retains the activity of ubiquinol reductase^27^, indicating that the exact role of the heme in this electron-transport protein is not fully understood. In the study, we have determined the structure of QFR in *D*. *gigas* with all the cofactors at a resolution of 3.6 Å. The overall architecture of this QFR comprises the subunit A (FAD-binding protein), the subunit B (iron-sulfur protein) and the subunit C (membrane-embedded protein). The relative locations of the redox cofactors in *D*. *gigas* and *W*. *succinogenes* QFRs are similar, suggesting that the electron transfer pathway, heme *b*_L_-heme *b*_H_-[3Fe:4S]-[4Fe:4S]-[2Fe:2S]-FAD, might be generally similar in *D*. *gigas* QFR to that in *W*. *succinogenes*. However, one menaquinone molecule was identified near the helix 1 in the subunit C. In *E*. *coli*, QFR contains the distinct sequential redox cofactors of FAD-[2Fe:2S]-[4Fe:4S]-[3Fe:4S]-Q_P_-Q_D_ without heme(s) in the two-chain membrane-embedded subunit. Here, Q_P_ was proposed to be the quinol-oxdizing site^10,28^, and Q_D_ might not be involved in the electron transfer pathway^11^. These observations and implications indicate that *D*. *gigas* QFR possesses a different electron transfer pathway from that in *E*. *coli*.

The superimposed structures of subunits C from *D*. *gigas* QFR and subunits C and D in *E*. *coli* QFR indicate that the *b*_L_ and *b*_H_ hemes in *D*. *gigas* QFR are structurally near Q_P_ and Q_D_ in *E*. *coli* QFR (Fig. S8), but the distance of closest approach between the *b*_L_ and *b*_H_ hemes (~4.5 Å) is much less than that between Q_P_ and Q_D_ (~25 Å), indicating that a direct electron transfer is more reasonable between the *b*_L_ and *b*_H_ hemes of *D*. *gigas* QFR than between Q_P_ and Q_D_ in *E*. *coli* QFR. If so, Q_P_ and Q_D_ might function more or less independently in mediating proton-coupled electron transfer events across the membrane in *E*. *coli*. If the *b*_L_ heme in *D*. *gigas* QFR is involved in the E pathway, the menaquinol substrate-binding site (MQ1) is expected to be near this heme (<14 Å, the edge-to-edge distance for direct electron transfer)^29^. The first possibility for the binding site of this putative menaquinol (MQ1) might be the same position as that previously proposed for QFR in *W*. *succinogenes*, near Glu-C60 in *D*. *gigas* QFR, but there is no extra electron density in this region. Furthermore, the proposed binding cavity for the head group of this putative menaquinol is deformed, and the other cavity for the hydrophobic tail is more hydrophilic in *D*. *gigas* QFR, indicating that *D*. *gigas* QFR might have a menaquinol-binding site different from that of *W*. *succinogen*es QFR. Instead, the head group of the fully reduced menaquinol substrate might bind at the more hydrophilic cavity near the *b*_L_ heme without the involvement of Glu-C60 in *D*. *gigas* QFR.

Interestingly, in the present study, we have located a menaquinone (MK) near the top of C-helix1. The distance (~13 Å) between this presumably fully oxidized quinone molecule and the *b*_L_ heme in *D*. *gigas* QFR is reasonable to allow a direct electron transfer from the *b*_L_ heme to this menaquinone, and subsequently to the Fe-S clusters in the subunit B and eventually to the fumarate in subunit A.

Although the distance ~28 Å between MK and [3Fe:4S] is too large for the direct electron transfer, there are many aromatic residues along the path between the two cofactors. These aromatic residues, including Trp-C36, Phe-C171, Phe-C189, Tyr-C172, Phe-C180 and Tyr-C178, might serve as the electron carriers to relay the long-distance electron transfer, as aromatic residues are known conduits for long-range electron transfer^30^.

We propose that MK, the bound menaquinone molecule, might serve as a coupling site to gate the proton-coupled electron transport across the membrane in *D*. *gigas* QFR, at least for one of the two reducing equivalents from the menaquinol substrate. When MK accepts the electron from the *b*_L_ heme, it becomes the semi-oxidized menaquinone (MKH). Subsequently, as an electron is transferred to the [3Fe:4S] cluster, the side-chain O–H proton of the Tyr-C63 that is hydrogen-bonded to the semi-menaquinone anion, can undergo synchronous movement in concert with the electron transfer to the cytoplasmic side of subunit C. Following this proton-coupled electron transport, the Tyr-C63 can acquire a proton from the periplasm *via* a water channel or proton donors occupying a proton-transfer pathway on the periplasmic side of subunit C, and the fully oxidized menaquinone MK is restored. In principle, the MK can cycle between MK and MKH again, and in two cycles transfers the two reducing equivalents of the menaquinol substrate to subunit B across the membrane *via* the high and low potential hemes in the QFR of *D*. *gigas*. However, based on the X-ray structure, we surmise that it is more likely that one of the two reducing equivalents of the menaquinol substrate is transferred directly from the high-potential heme *b*_H_ without the involvement of MK (Fig. 7).

The bifurcation of the electron flow, namely the transfer of the two reducing equivalents upon the oxidation of the quinol substrate through two heme centers, is well known and exists in many electron-transport proteins^31,32^. As the edge-to-edge distances relevant for electron transfer here are shorter than 14 Å^29^, the *b*_L_ heme in *D*. *gigas* QFR is far from the [3Fe:4S] cluster (~28 Å). However, it is near the bound MK (~13 Å), indicating that electrons generated by the oxidation of the menaquinol substrate at the MQ1 site can pass through the *b*_L_ heme. In this scenario, one of the two reducing equivalents of the menaquinol substrate is initially shuttled to the *b*_H_ heme. This electron could be subsequently transferred to the MK via the *b*_L_ heme during the second half of the turnover cycle, or it could be transferred directly to the [3Fe:4S] in subunit B.

To avert the buildup of an electrostatic potential across the membrane, the transmembrane electron transfers must be accompanied by coupled proton transfers during the oxidation of the menaquinol substrate and the reduction of fumarate to succinate on opposite sides of the membrane. The proton-transfer pathway(s) are still not clearly elucidated. The proton-active residues, glutamates and histidines, and protein-bound water molecules are considered the most commonly to be involved in the proton transfers^33^. Examination of the QFR structure from *D*. *gigas* shows two possible proton acceptors, Glu-C60 and His-C38, near the bound MK molecule, suggesting the possibility of two potential proton-transfer pathways, A and B, in *D*. *gigas* QFR (Figs S9 and S10). As shown in Fig. 6, in the proton-transfer pathway A, the distance between the O–H group of Tyr-C63 in the hydrogen bond of the partially oxidized MK, i.*e*., the semi-menaquinone anion (MKH), and the carboxyl group of Glu-C60 is ~8.8 Å, which is too large for a direct proton transfer (4 to 5 Å)^33^. But the proton transfer might be mediated by water molecules in a protein water channel, despite that no bound waters are observed in our QFR structure. Glu-C60 in *D*. *gigas* corresponds to Glu-C66 that was earlier suggested to be involved in the menaquinol binding or in proton transfer in *W*. *succinogenes*^13^. Thus, in the proton-transfer pathway A, the proton is transferred from the periplasmic space *via* Glu-C60 to the side-chain of Tyr-C63 after reoxidation of the MKH. In the proton-transfer pathway B (the transmembrane proton-transfer pathway), the distance between His-C38, the axial ligand of the *b*_L_ heme, and the MKH in the hydrogen bond with the Tyr-C63 hydroxyl group, is ~12.8 Å. Again, water molecules are thus necessary to mediate the transfer of the proton from the MKH to the His-C38 in this proposed proton transfer pathway. From the analysis of glutamate and histidine residues in the QFR structure of *D*. *gigas* (Fig. S10b), the only possible proton acceptor from His-C38 is Glu-C164, so during the proton-coupled electron transfer mediated by MK, the transfer of the proton might be mediated by iron-coordinated His-C38 (and/or the *b*_L_ ring C-propionate) of heme *b*_L_ to Glu-C164. From Glu-C164, the subsequent proton acceptor might be Glu-C193 (Fig. S10b), which is a specific proton acceptor unique to *D*. *gigas* QFR. This proton acceptor is not found in *W*. *succinogenes* and *E*. *coli* QFRs (Fig. S4), indicating that *D*. *gigas* QFR might adopt a unique pathway, His-C38~Glu-C164~Glu-C193~cytoplasm, to transfer the redox-linked or coupled proton-transfers across the inner membrane to the cytoplasm (Fig. S10b). Note that His-C38 and Glu-C164 in QFR of *D*. *gigas* correspond structurally with His-C44 and Glu-C180 in *W*. *succinogenes* QFR. These two residues were identified to be critical for proton transfers in *W*. *succinogenes* QFR^6,14,34^. The transmembrane proton pathway between His-C38 and Glu-C164 might thus be similar to QFR in *W*. *succinogenes*, but the proton transfer between Glu-C164 to Glu-C193 seems to be unique to *D*. *gigas* QFR.

In summary, no suitable cavity near Glu-C60 in the C subunit has been identified for binding of the head group of the menaquinol substrate in the QFR structure of *D*. *gigas*, in contrast to the head-group binding cavity previously proposed for menaquinol in *W*. *succinogenes* QFR. The head group of the fully reduced menaquinol substrate molecule might bind instead to the more hydrophilic cavity close to the *b*_L_ heme in *D*. *gigas* QFR. Since in this study crystals of the QFR are obtained on the enzyme isolated from the *D*. *gigas* grown under fumarate, the product menaquinone should not be associated with the enzyme in the protein preparation, and in fact, we have not detected one. Instead, we have identified a new bound menaquinone near helix 1 and heme *b*_L_ in the QFR structure of *D*. *gigas*. We propose that this bound menaquinone (MK) serves as a novel redox cofactor, or a coupling site to gate the proton-coupled electron transport across the membrane in *D*. *gigas* QFR. In addition, a newly identified potential proton acceptor, Glu-C193, might be important in the redox-linked proton transfer across the membrane in the *D*. *gigas* QFR. On the basis of the QFR structure of *D*. *gigas* in a complex with the bound menaquinone, as well as comparison with the QFR structures of *W*. *succinogenes*, *E*. *coli* and *A*. *suum*, we have clarified the roles of the two *b* hemes in the electron-bifurcation pathway in the menaquinol oxidation and delineated the mechanism of proton-coupled pathways from the periplasm across the membrane during the transfers of the reducing equivalents from the quinol to fumarate in *D*. *gigas* QFR (Fig. 7). Finally, in light of this study, we may perhaps understand how the QFR in *E*. *coli* can function to mediate electron transfers without any *b* hemes. With two bound menaquinones, these cofactors can participate in the bifurcation of the electron flow from the quinol substrate and also serve as the coupling sites to gate the proton-coupled electron transport across the membrane exploiting the mechanism that we are proposing here for at least one of the reducing equivalents in *D*. *gigas* QFR.

## Methods

### Cell growth and preparation of cell extracts

*Desulfovibrio gigas* cells were grown in a fumarate/sulphate medium to obtain a large quantity of cells. The cells were grown over a period of ~24 h (OD_600_ ≈ 1.0) at 37 °C with a ten-fold transformation in a 600 or 2000 L fermenter, producing 300 g or 1 kg of cells, respectively. The cultured cells were suspended in a buffer with Tris-HCl (20 mM, pH 7.6), and disrupted twice with a high-pressure homogenizer (AVESTIN ElulsiFlex-C3, 1500 psi) at room temperature. The disrupted cells were collected on ice immediately. After cell breakage, the suspension was centrifuged (KUBOTA 6930, 8000 rpm, 40 min) at 4 °C. The cell extract was concentrated further with an ultra-centrifuge (HITACHI CP70MX, 40,000 rpm, 2 h) at 4 °C. The supernatant was discarded; the pellet was washed (membrane fraction; black), twice with a buffer (volume 10 times) containing Tris-HCl (20 mM)/EDTA (1 mM), and once with 10 volumes of Tris-HCl (20 mM)/glycerol (10%). When the pellet turned from black to brown, the proteins were extracted twice from the pellet with a buffer containing Tris-HCl (20 mM, pH 7.6), glycerol (10%) and DDM (4%) at 4 °C for 2 h. The supernatant separated by ultracentrifugation (40,000 rpm, 2 h) was then ready for further purification.

### Purification of QFR from *D. gigas*

All purification procedures were performed using the AKTA chromatography system (GE Healthcare) in a cold room (4 °C); all buffers used contained 0.1% DDM and were adjusted to pH 7.6. Crude extract was applied to a DEAE column (GE Healthcare, DEAE Sepharose Fast Flow) previously equilibrated with buffer A (Tris-HCl, 10 mM, pH 7.6), glycerol (10%) and DDM (0.1%, *w/v*). The column was eluted with a linear gradient (0 to 500 mM) of sodium chloride supplemented in buffer A, and the QFR was present in the fraction corresponding to sodium chloride (180 to 200 mM) of the eluted buffer. The QFR fraction was subjected to dialysis to decrease the ionic strength in buffer A. This fraction was then loaded onto a Q-sepharose column (GE Healthcare, Q Sepharose Fast Flow) and the protein faction was again eluted with NaCl (~200 mM). As there were some minor contaminations in this step, we performed a final size-exclusion column chromatography (GE Healthcare, Superdex 200 10/300 GL, equilibrated with 20 mM Tris buffer, pH 7.6, containing 10% glycerol and 0.1% DDM) to remove these contaminants. The protein fraction was concentrated in an ultrafiltration cell (Amicon) with molecular-weight cutoff 100 kDa. The purity of the fraction was examined with SDS-PAGE and the UV-visible spectrum (Fig. S3a).

### Crystallization and X-ray data collection

Purified *D*. *gigas* QFR was concentrated (10 mg/mL) without glycerol for the crystallization experiment. Initial crystal screenings were performed with a crystallization robot (Mosquito Crystal, TTP Labtech) with the hanging-drop vapor-diffusion method on mixing the protein sample (100 nL) with a reservoir solution (100 nL) with several screen kits. Small crystals were observed in one condition containing PEG 4000 (15%), NaCl (200 mM) in a sodium acetate buffer (100 mM, pH 4.6) at 18 °C after two weeks. Crystals of *D*. *gigas* QFR were then transferred to a crystallization buffer containing glycerol (20%) for cryoprotection and were immediately frozen in liquid nitrogen.

The X-ray diffraction experiments were performed with photons of wavelength 0.9 Å on TLS beamlines BL13C, BL15A and TPS 05 A at the NSRRC (Taiwan) and BL44XU and BL12B2 at SPring8 (Japan). The screened crystals initially diffracted to only approximately 10 Å. Protein crystals were then further optimized using hanging drops consisting of equal volumes of protein solution (1 μL) and a reservoir solution (1 μL) equilibrated against a reservoir solution (150 μL) containing NaCl (100 mM), PEG 4000 (28%) and Tris buffer (100 mM, pH 8.5) at 18 °C for two weeks. The improved larger crystals grew to a final size 0.2 × 0.2 × 0.1 mm^3^ (Fig. S3b). The best complete data set at resolution 3.6 Å (although some diffractions were observed at 3.5 Å) was collected at BL44XU after a number of crystals were screened simultaneously at BL12B2 of SPring-8. The data were processed with *HKL2000*. All X-ray data statistics are summarized in Table 1.

**Table 1:** Statistics of crystal diffraction and structure refinement.

**I. Crystal data**	**QFR (PDB entry 5XMJ)**
Wavelength (Å)	0.9
Temperature (K)	100
Unit-cell parameters (Å)	*a* = 112.14, *b* = 131.77, *c* = 195.43
Resolution range (Å)	30–3.6 (3.73–3.6)^a^
Space group	*P*2_1_
Unique reflections	63889 (6426)^a^
Completeness (%)	98.5 (99.4)^a^
*I*/σ_*I*_	19.2 (2.2)^a^
*R*_pim_	0.065 (0.497)^a^
CC_1/2_	0.996 (0.725)^a^
Average redundancy	4.0
Mosaicity (°)	0.92
No. of ABC complexes per A.U.	4
**II**. **Refinement results**	
Resolution range (Å)	30–3.6
^b^*R*_work_ (%)	25.22
^c^*R*_free_ (%)	30.84
Rmsd bond length (Å)	0.012
Rmsd bond angles (°)	1.52
Total number of non-hydrogen atoms	34118
Total number of protein atoms (non-hydrogen)	33360
Total number of ligand atoms	758
Total protein residues	4296
Ramachandran plot	
Most favored (%)	89.65
Allowed (%)	10.35
Disallowed (%)	0
Rotamer outliers (%)	0.37
Clashscore/Molprobity score	25.45/2.45

^a^The highest resolution shell.

^b^*R*_work_ = Σ_hkl_ | F_o_ − F_c_ |/Σ_hkl_ F_o_, where F_o_ and F_c_ are the observed and calculated structure factor amplitudes of reflections.

^c^*R*_free_, calculated the same as *R*_work_ but from a test set containing 5% of data excluded from the refinement calculation.

### Structure determination

The processed data were reduced from averaged intensities to mean amplitudes with *TRUNCATE* in *CCP4* suite^35^. The initial phase was determined with the molecular replacement method using *MOLREP*^36^ in *CCP4* suite with the structure of QFR from *W*. *succinogenes* (PDB entry 2BS2, sequence identity ~50%) without redox cofactors as the search model. Two homo-dimers formed by four sets of the hetero-trimeric complex (subunits A, B and C) were found in the asymmetric unit. Iterative cycles of model building and refinement using *COOT*^37^ and *PHENIX*^38^, respectively, were performed. During the refinement, NCS restraints, secondary-structure restraints and the *B*-group refinement were applied. The redox cofactors, including hemes, iron-sulphur clusters, FAD and fumarate, were built based on electron density maps (2*F*_o_ − *F*_c_ and *F*_o_ *−* *F*_c_). Detergent (DDM) molecules and the menaquinone (MK) at the MQ2 site were finally fitted into appropriate positive *F*_o_ − *F*_c_ electron density maps. Iterative cycles of the model adjustment and refinement were performed in the last step to decrease the final *R*_work_/*R*_free_ factors to 25.22%/30.84% at resolution 3.6 Å. An attempt to extend the resolution of refinement to 3.5 Å was made, which increased both the *R*_work_*/R*_free_ values because of the insufficient data quality beyond 3.6 Å. The refined structure of QFR of *D*. *gigas* was validated with *MolProbity*^39^. The rotamer outlier, clashscore and *MolProbity* score are 0.37%, 25.45 and 2.45, respectively. The calculations of root-mean-square deviations of the angles, bonds and dihedral and improper angles showed the satisfactory stereochemistry. The figures were prepared with *PyMOL*^40^.

### Accession Codes

The structural factors and coordinates have been deposited in the RCSB Protein Data Bank. The PDB code is 5XMJ.

## Electronic supplementary material

Supplementary Figures
