# Supplementary material for: Structural insights into the electron/proton transfer pathways in the quinol:fumarate reductase from Desulfovibrio gigas
Source: Sci Rep. 2018 Oct 8;8:14935. doi: 10.1038/s41598-018-33193-5 (PMC6175931; doi:10.1038/s41598-018-33193-5)
Supplement: Supplementary file 1 — Supplementary Figures [file 41598_2018_33193_MOESM1_ESM.pdf]

Supplementary Figures

Structural insights into the electron/proton transfer pathways in the quinol:fumarate reductase from *Desulfovibrio gigas*

Hong-Hsiang Guan[1†], Yin-Cheng Hsieh[1†], Pei-Ju Lin[1,2], Yen-Chieh Huang[1], Masato Yoshimura[1], Li-Ying Chen[1,3], Shao-Kang Chen[1,3], Phimonphan Chuankhayan[1], Chien-Chih Lin[1], Nai-Chi Chen[1], Atsushi Nakagawa[5], Sunney I. Chan[6,7], and Chun-Jung Chen[1,3,4*]

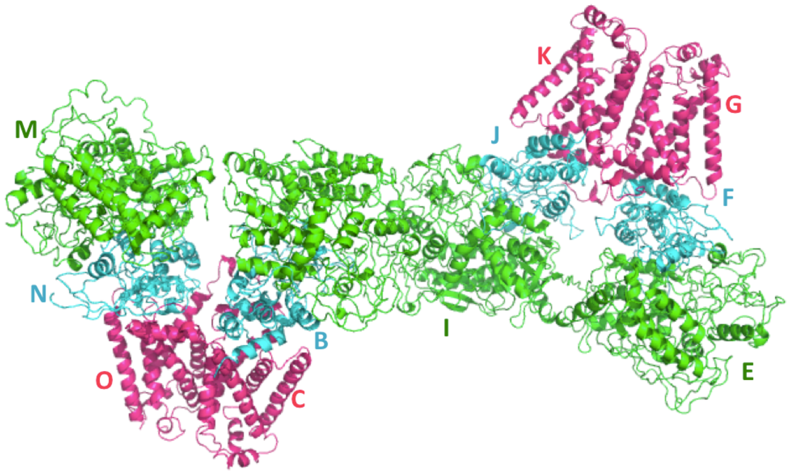

Structure 1/Structure 2	Buried surface area (Å^2)	Portion (%)
Subunit C/O	1327/1353	9.4/9.7
Subunit A/M	468/475	1.9/2.2
Subunit B/N	negligible	negligible
Subunit G/K	1248/1216	8.7/8.5
Subunit E/I	546/547	2.5/2.4
Subunit F/J	negligible	negligible
Subunit A/I	386/379	1.7/1.7
Subunit A/B	2679/2839	12.1/21.8
Subunit B/C	1634/1610	12.5/11.4

Calculated by PISA

Fig. S1. Buried surface areas in the *D. gigas* QFR. The buried surface area between individual subunits and the corresponding portions related to the total surface based on the determined QFR structure of *D. gigas* are calculated by *PISA* and listed in the table. FAD-binding proteins, iron-sulphur proteins and membrane-embedded proteins are colored green, cyan and pink, respectively.

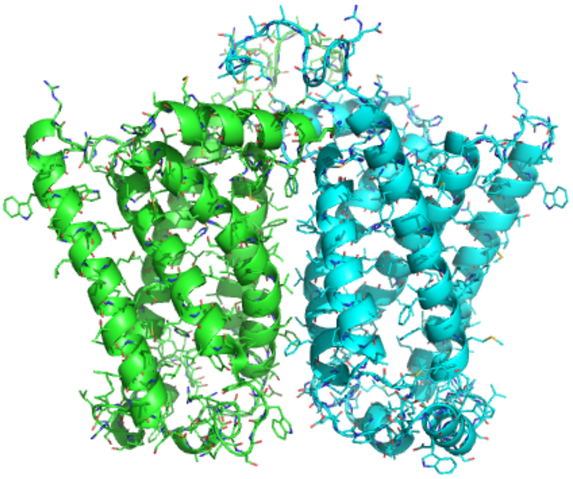

0.219
in Complex Formation Significance Score (CSS).
CSS ranges from 0 to 1 as interface relevance to complex formation increases.
Achieved CSS implies that the interface plays an auxiliary role in complex formation

C subunit/O	Distance (Å)	C subunit/C
O:ARG 85[NH1]	3.37	C:GLN 91[OE1]
O:GLN 91[OE1]	3.49	C:ARG 85[NH1]
O:GLY 151[O]	3.71	C:THR 135[OG1]

Fig. S2. The intermolecular interface between the two subunits C. Two adjacent subunits C are colored as green and cyan, respectively. The weak hydrogen bonds, with donor-acceptor distances of 3.2 – 4.0 Å, in the interface are shown in the table.

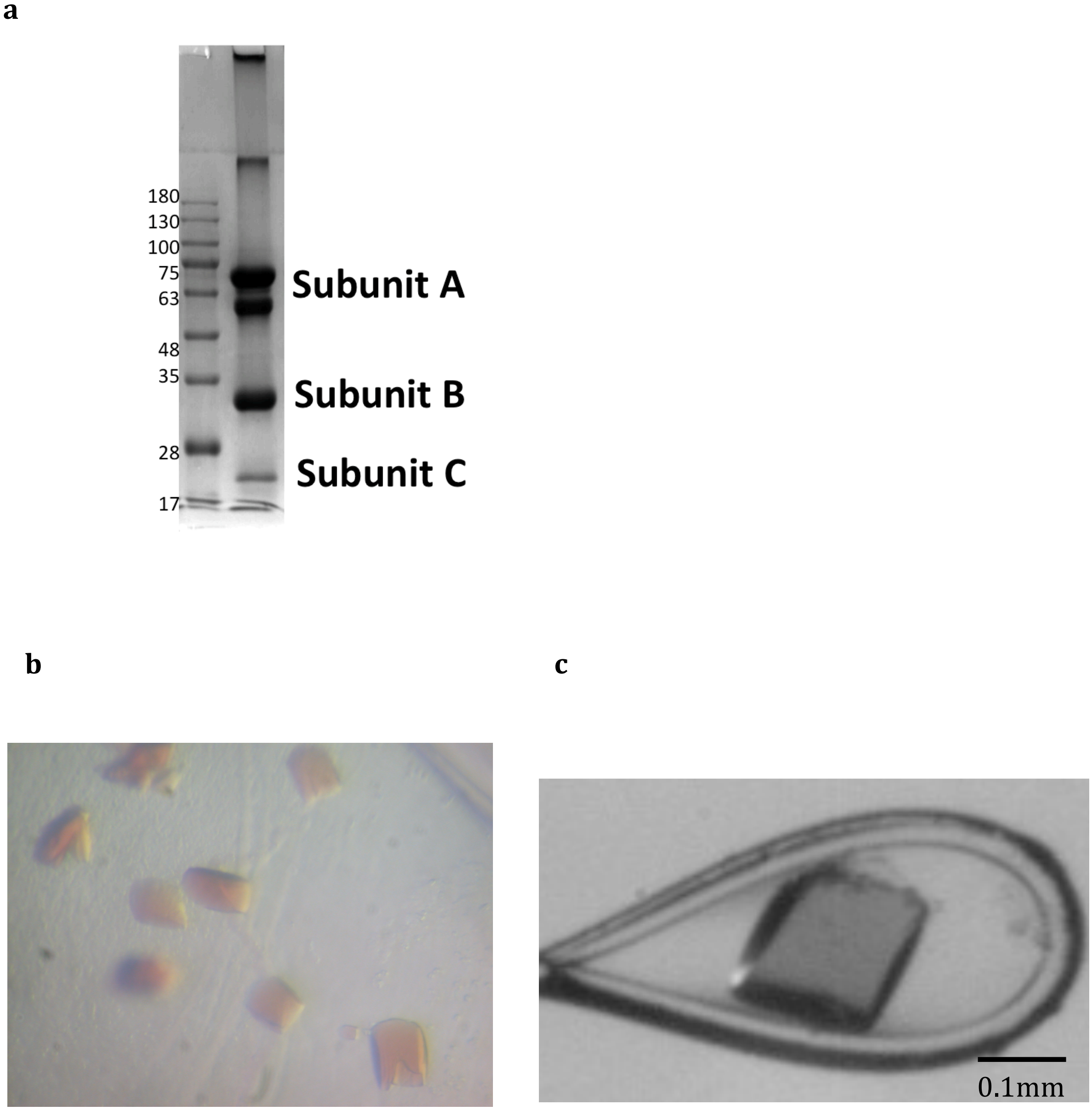

Fig. S3. Purification and crystallization of QFR from *D. gigas*. (**a**) SDS-PAGE (10%) of purified protein from the Superdex-G200 (Superdex 200 10/300 GL) column. Lane 1, molecular-weight size marker and 2, purified QFR. Subunits A, B and C are indicated. The band below that of the subunit A is the proteolytic fragment of the subunit A and validated by the liquid chromatography-tandem mass spectrometry (LC-MS/MS) (**b**) The initial QFR crystals. (**c**) The optimized crystal of QFR on the loop was used for data collection.

a Subunits A

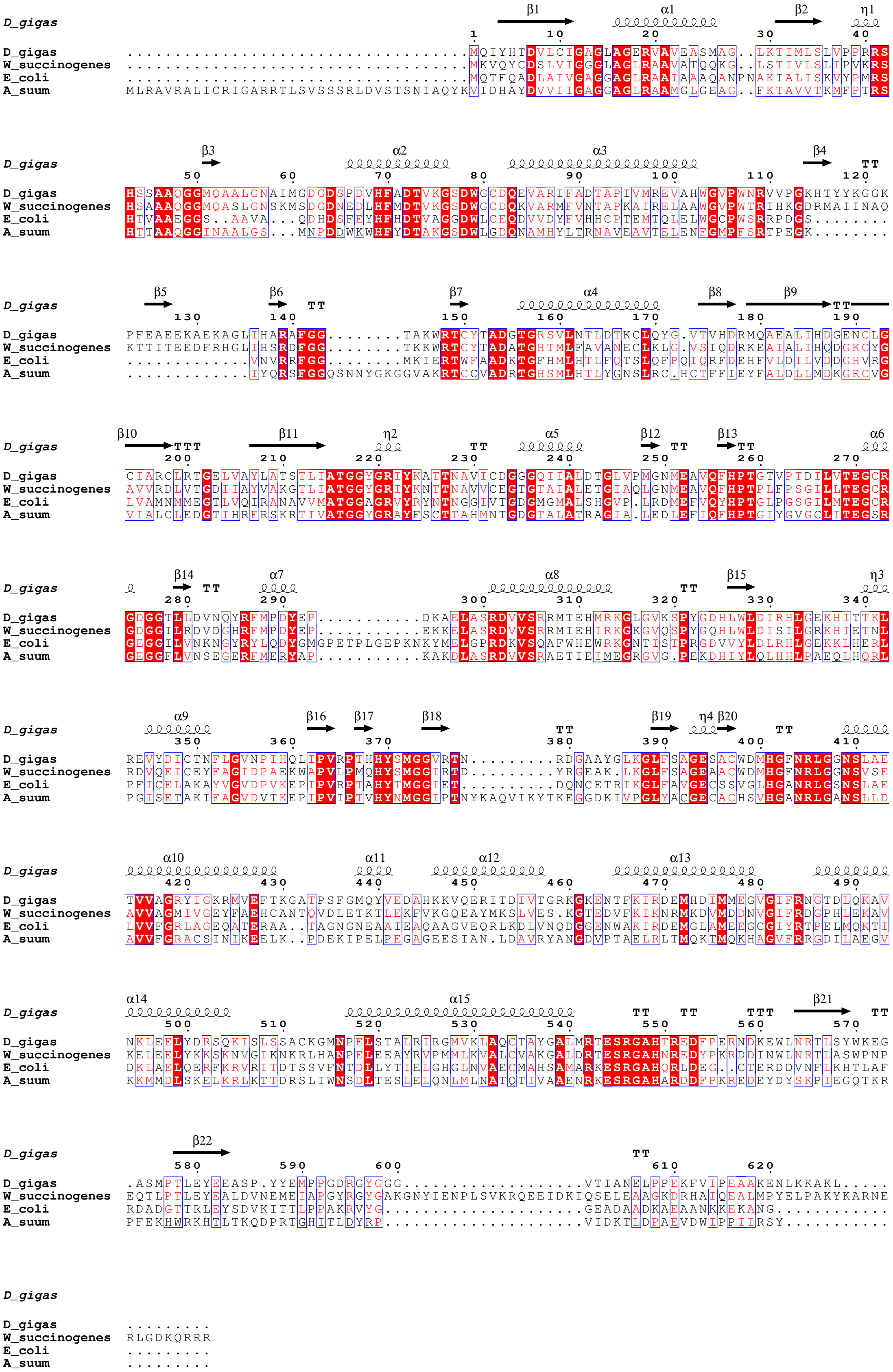

b Subunits B

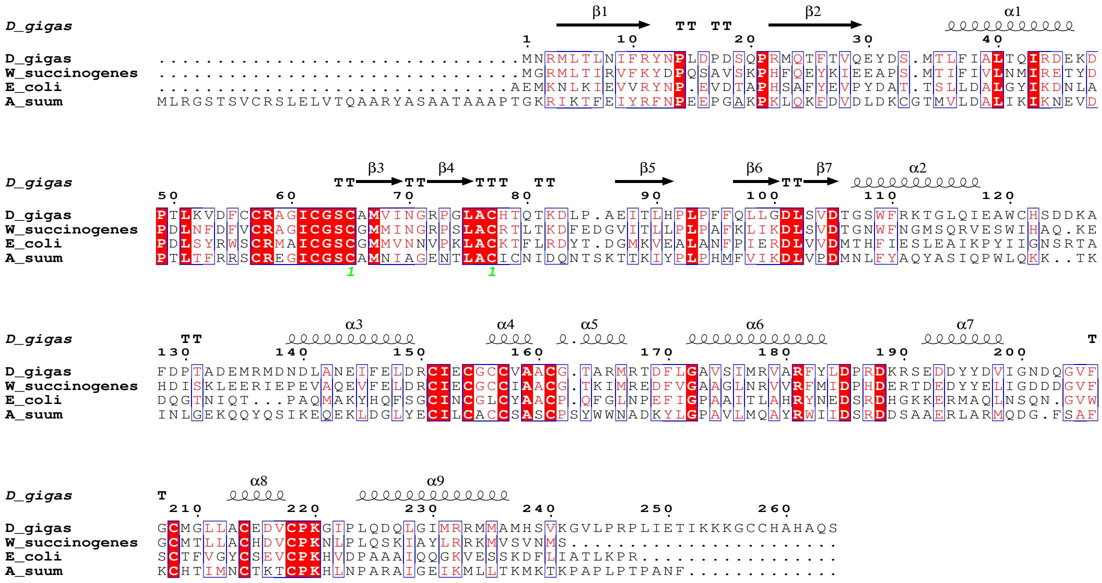

c Transmembrane domains

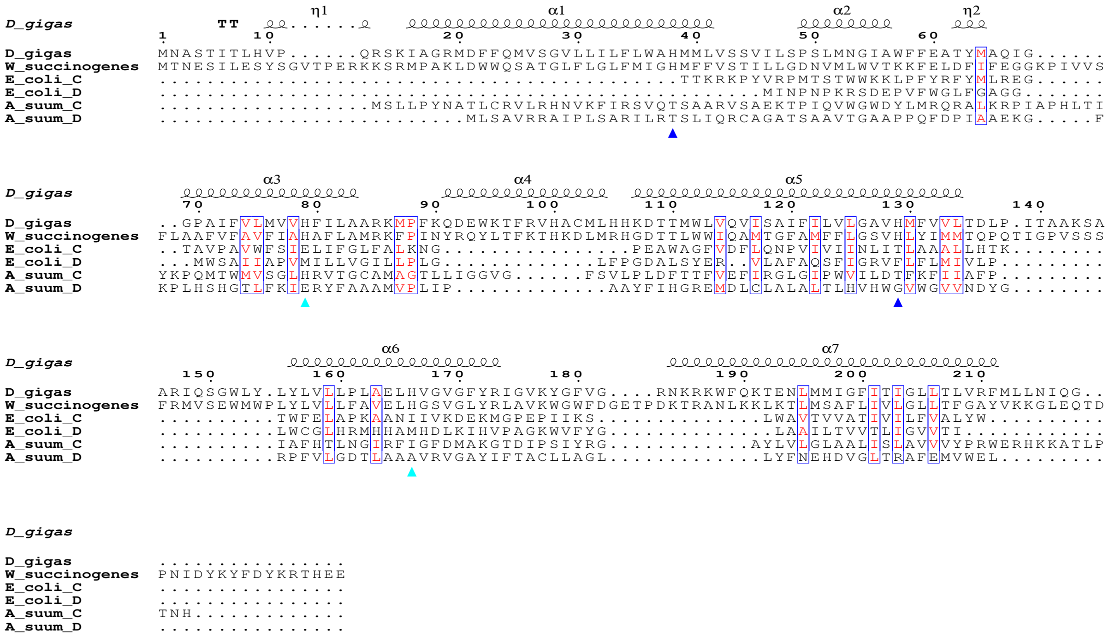

Fig. S4. The sequence alignment of distinct subunits of known structures from *D. gigas*, *W. succinogenes*, *E. coli*. and *A. Suum* (**a**) Subunits A (flavoproteins) **(b)** Subunits B (iron-sulphur proteins) and **(c)** Transmembrane domains (subunits C from *D. gigas*, and *W. succinogenes* and subunits C and D from *E. coli* and *A. suum)*. The alignment was performed with *ClustalW* (http://embnet.vital-it.ch/software/ClustalW.html) and *ESPript* (http://espript.ibcp.fr/). α and β denote the α-helix and β-strand respectively. Histidine pairs coordinate hemes in the C subunit from *D. gigas* are labeled as blue and cyan triangles.

a **b**

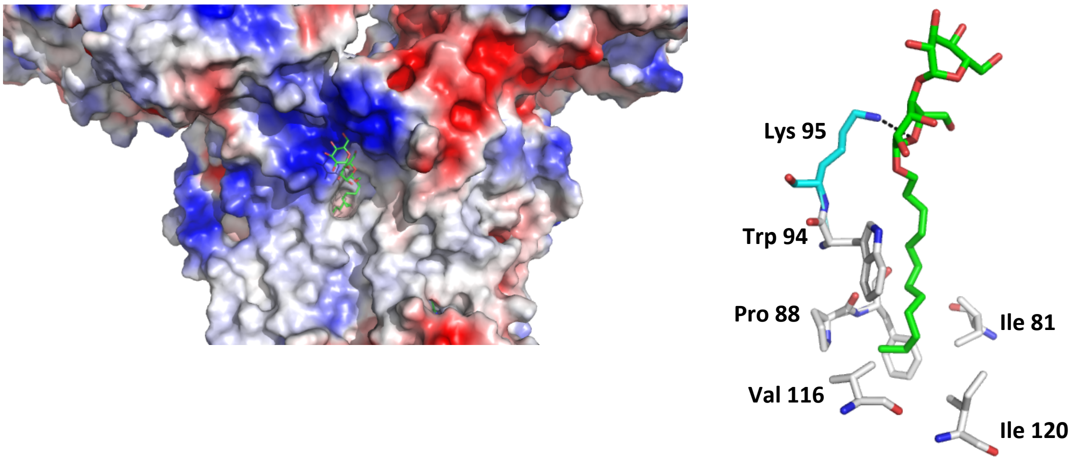

Fig. S5. A bound detergent n-Dodecyl β-D-maltoside (DDM) in the subunit C of *D. gigas*. (**a**) The QFR structure from *D. gigas* is shown with the electrostatic surface potential plot. The hydrophobic tail of DDM is bound in a hydrophobic pocket and the DDM is shown as green stick. (**b**) The bound DDM forms a hydrogen bond shown as dot lines with Lys-C95 colored as cyan and the hydrophobic tail of DDM is accommodated in the hydrophobic pocket colored as light gray. The hydrophobic residues formed the hydrophobic pocket are labeled.

a

FAD-binding protein

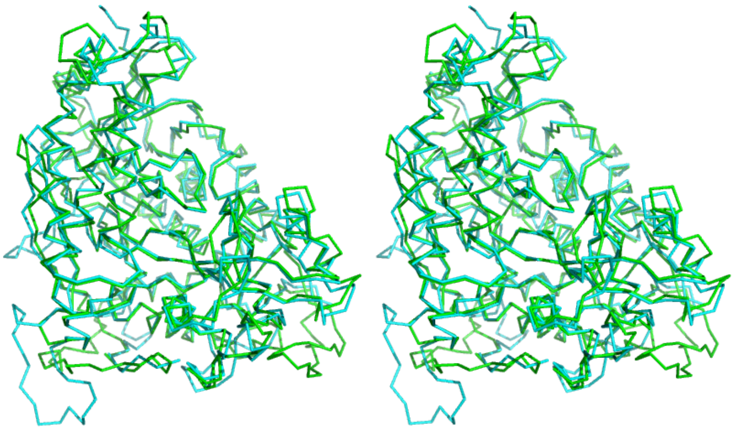

b

Iron-sulphur protein

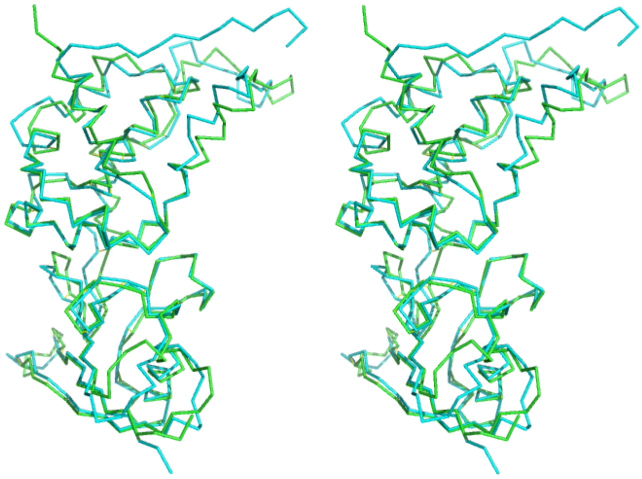

c

Membrane-embedded proteins

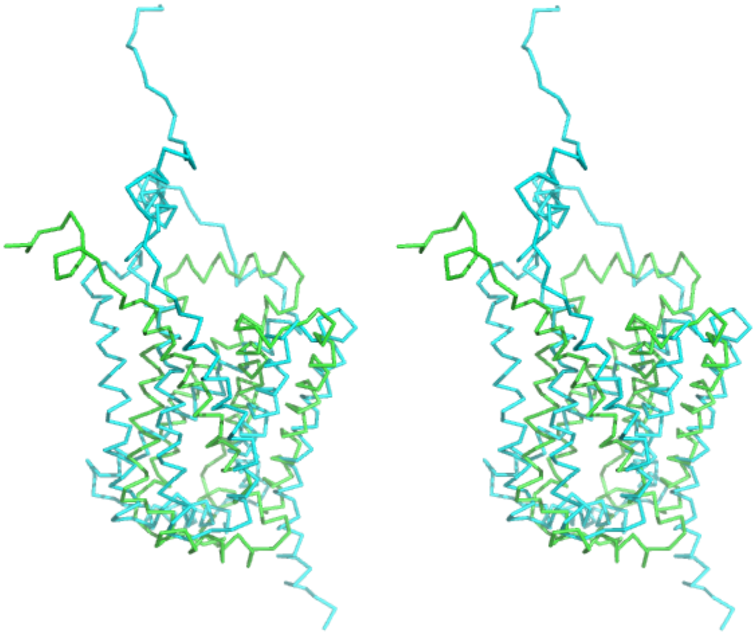

Fig. S6. Stereo views of the superimposed structures of FAD-binding proteins, iron-sulphur proteins and membrane-embedded proteins between *D. gigas* and *A. suum* QFRs respectively. (a) FAD-binding proteins, **(b)** iron-sulphur proteins, and **(c)** membrane-embedded proteins. *D. gigas* and A. *suum* QFRs are shown as green and cyan ribbons respectively.

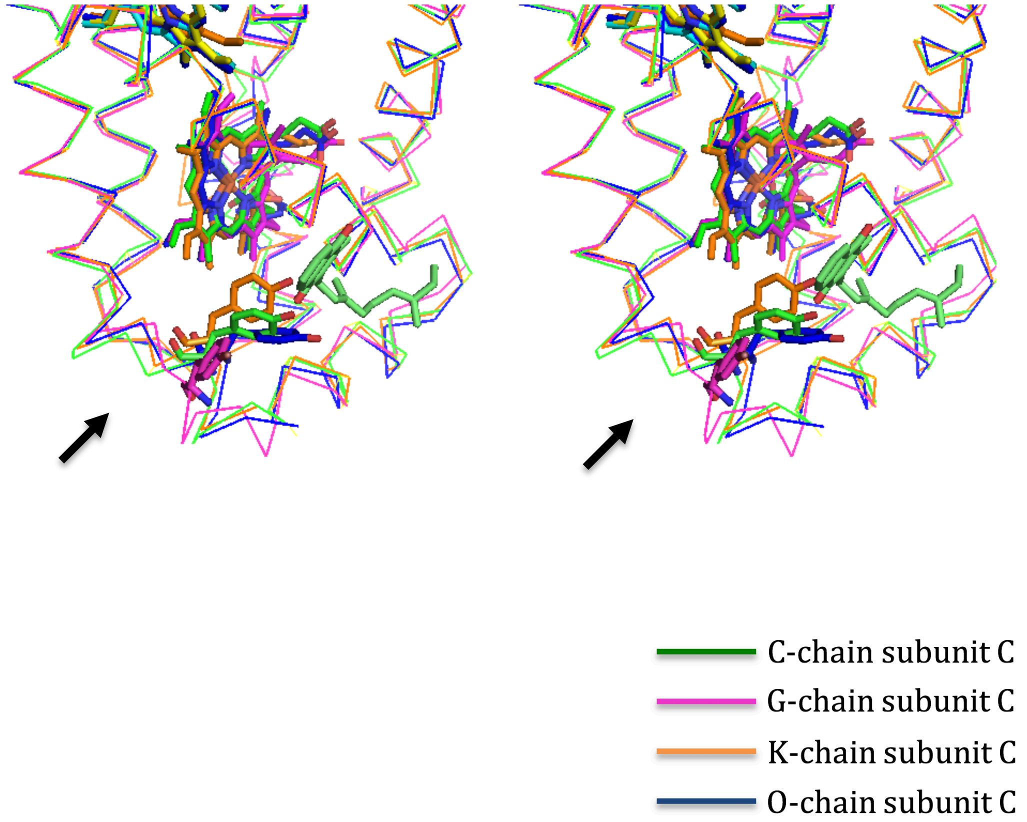

Fig. S7. A stereo view of superimposed structures of four subunits C near the menaquinol-binding sites of *D. gigas* QFR. The bound menaquinol molecule and the interacted Tyr-C63 of four chains in the asymmetric unit are shown as sticks. The four hemes are shown as green, orange, pink and blue sticks. The flexible short loop between helices 2 and 3 is indicated by the black arrow.

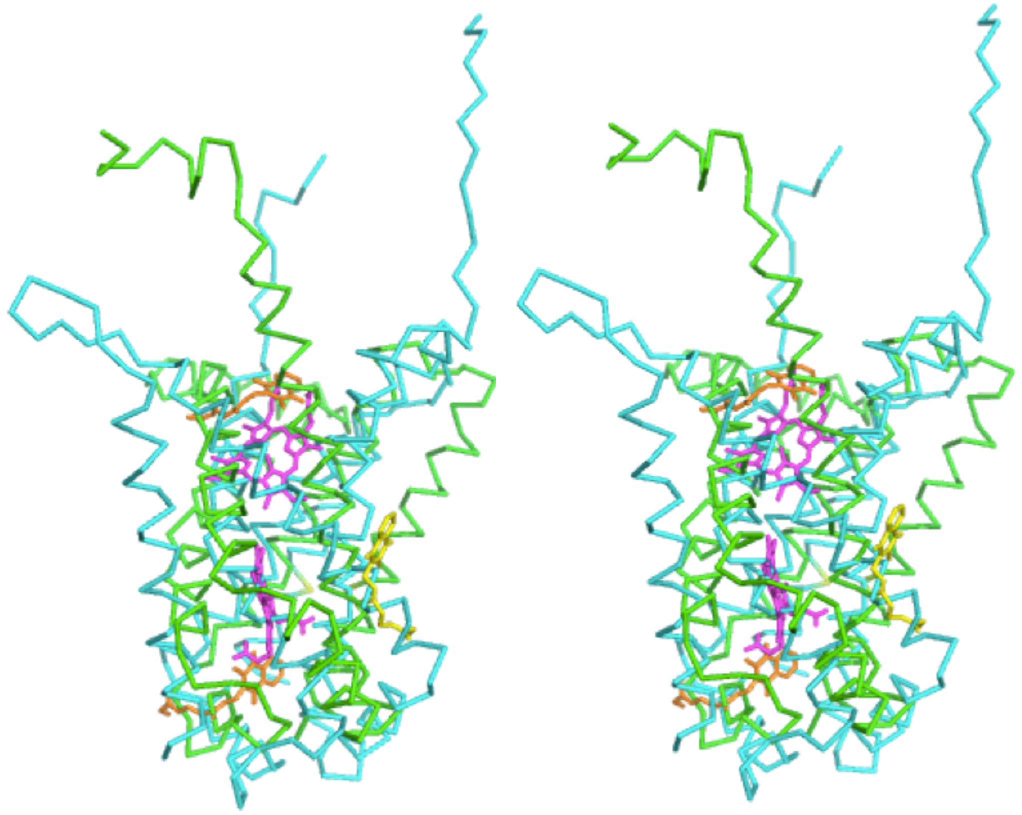

Fig. S8. A stereo view of superimposed membrane-embedded proteins of *D. gigas* and *E. coli* QFR. The membrane-embedded proteins of *D. gigas* and *E. coli* QFRs are shown as green and cyan ribbons respectively. The hemes and one bound menaquinone in *D. gigas* QFR are shown as pink and yellow sticks whereas the two menaquinone in *E. coli* QFR are shown as orange sticks.

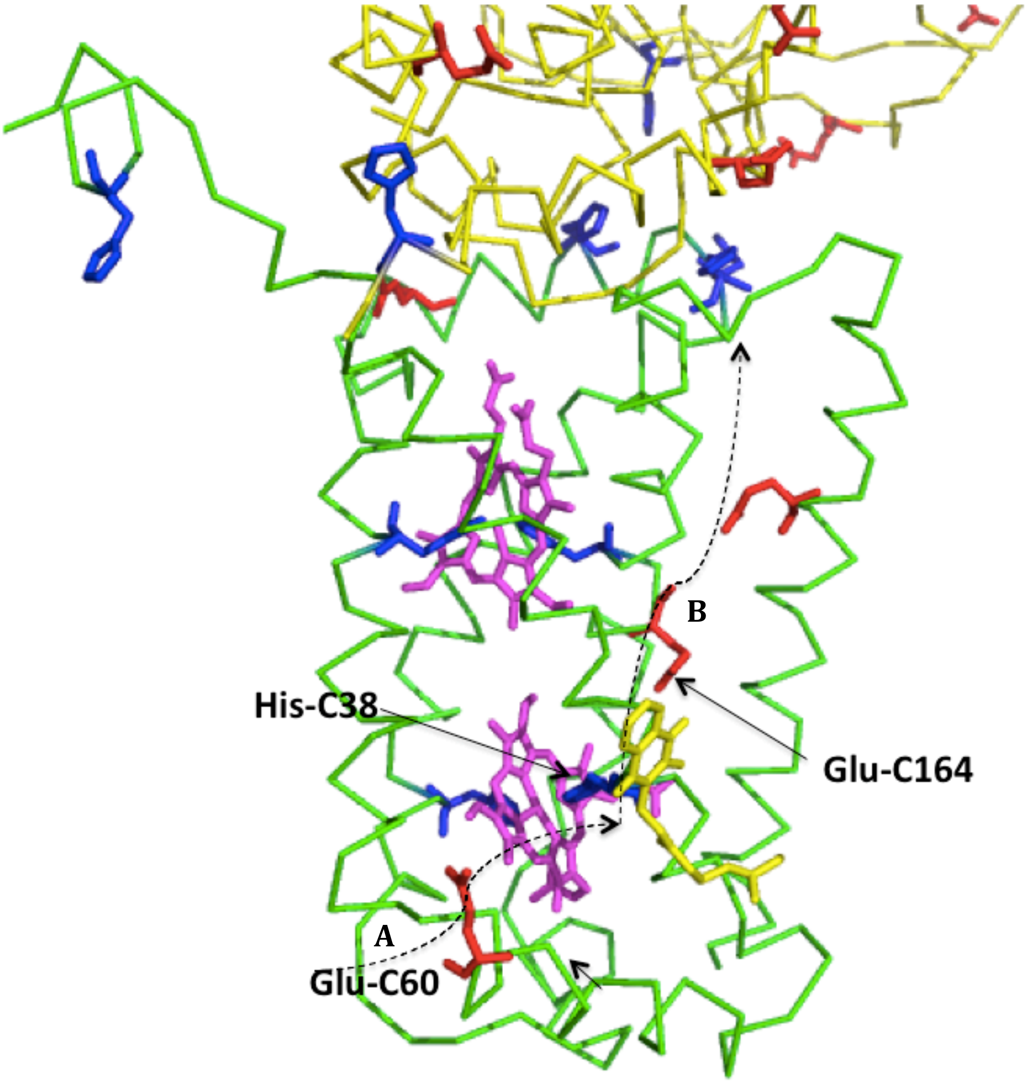

Fig. S9. The potential proton-transfer pathways in the subunit C of *D. gigas*. All potential proton transfer residues, glutamates and histidines, in the subunits B (yellow ribbon) and C (green ribbon) of *D. gigas* are shown. Glutamates and histidines in the subunit C are colored red and blue, respectively. The bound menaquinone is shown as the yellow stick. The possible proton pathways, A and B, in the subunit C are shown as dash lines and three critical residues near the bound menaquinone for proton transfer are indicated by arrows and labeled. Hemes are colored pink.

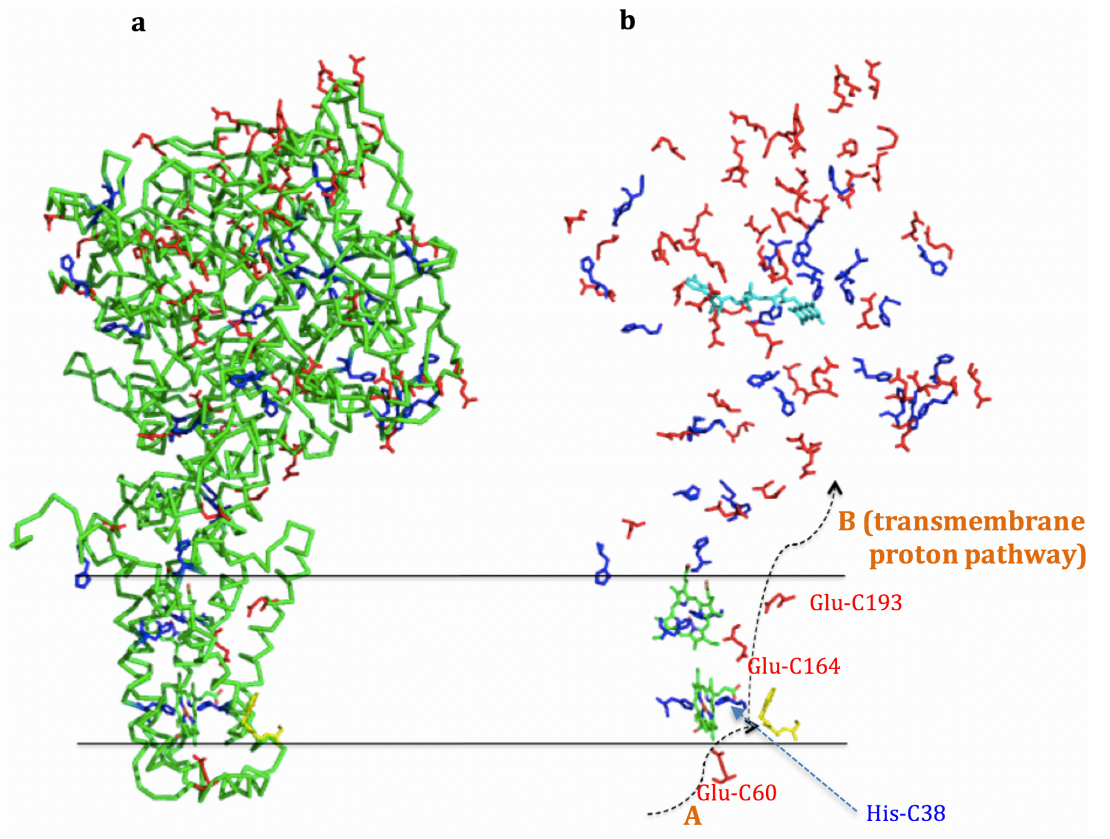

Fig. S10. All potential proton transfer residues, glutamates and histidines, in the QFR of *D. gigas* are shown. (**a**) Glutamates and histidines are colored red and blue respectively and the QFR protein backbone is shown as green ribbon. (**b**) Only glutamates and histidines are shown as red and blue respectively. The possible proton pathways, defined as A and B, in the QFR are shown as dash. The destination of the electron transfer, fumarate bound in the subunit A, is shown as the cyan stick. The bound menaquinone is shown as the yellow stick. The inner membrane is indicated by the black solid line. The proposed key residues for the proton transfer are labeled.
